# Phytoplankton transcriptomic and physiological responses to fixed nitrogen in the California current system

**DOI:** 10.1371/journal.pone.0231771

**Published:** 2020-04-20

**Authors:** Irina N. Shilova, Jonathan D. Magasin, Matthew M. Mills, Julie C. Robidart, Kendra A. Turk-Kubo, Jonathan P. Zehr

**Affiliations:** 1 Department of Ocean Sciences, University of California Santa Cruz, Santa Cruz, California, United States of America; 2 Department of Earth System Science, Stanford University, Stanford, California, United States of America; 3 Ocean Technology and Engineering, National Oceanography Centre, Southampton, England, United Kingdom; Texas A&M University at Galveston, UNITED STATES

## Abstract

Marine phytoplankton are responsible for approximately half of photosynthesis on Earth. However, their ability to drive ocean productivity depends on critical nutrients, especially bioavailable nitrogen (N) which is scarce over vast areas of the ocean. Phytoplankton differ in their preferences for N substrates as well as uptake efficiencies and minimal N requirements relative to other critical nutrients, including iron (Fe) and phosphorus. In this study, we used the MicroTOOLs high-resolution environmental microarray to examine transcriptomic responses of phytoplankton communities in the California Current System (CCS) transition zone to added urea, ammonium, nitrate, and also Fe in the late summer when N depletion is common. Transcript level changes of photosynthetic, carbon fixation, and nutrient stress genes indicated relief of N limitation in many strains of *Prochlorococcus*, *Synechococcus*, and eukaryotic phytoplankton. The transcriptomic responses helped explain shifts in physiological and growth responses observed later. All three phytoplankton groups had increased transcript levels of photosynthesis and/or carbon fixation genes in response to all N substrates. However, only *Prochlorococcus* had decreased transcript levels of N stress genes and grew substantially, specifically after urea and ammonium additions, suggesting that *Prochlorococcus* outcompeted other community members in these treatments. Diatom transcript levels of carbon fixation genes increased in response to Fe but not to Fe with N which might have favored phytoplankton that were co-limited by N and Fe. Moreover, transcription patterns of closely related strains indicated variability in N utilization, including nitrate utilization by some high-light adapted *Prochlorococcus*. Finally, up-regulation of urea transporter genes by both *Prochlorococcus* and *Synechococcus* in response to filtered deep water suggested a regulatory mechanism other than classic control via the global N regulator NtcA. This study indicated that co-existing phytoplankton strains experience distinct nutrient stresses in the transition zone of the CCS, an understudied region where oligotrophic and coastal communities naturally mix.

## Introduction

Marine phytoplankton are responsible for about half of photosynthesis on Earth [1]. The growth and productivity of phytoplankton are constrained by the availability of critical nutrients, primarily nitrogen (N), phosphorus (P), and iron (Fe) [2–7]. Over wide areas of the ocean, N limits phytoplankton growth [7,8] because most phytoplankton cannot use the gaseous form, dinitrogen (N_2_). Instead they use a variety of other chemical forms of N, including organic forms such as urea, as well as inorganic forms such as nitrite (NO_2_^-^) and nitrate (NO_3_^-^), with preferences differing among phytoplankton [9–12]. Physical transport processes, remineralization, and other factors, often seasonal and region-specific, affect which N forms are available [9,13–16], which in turn affects species composition and trophic dynamics [17,18]. Much of our knowledge about phytoplankton communities comes from either oligotrophic ocean gyres or coastal regions, the latter of which typically have higher levels of available N. Less is known about boundary current systems where the two communities naturally mix, such as the transition zone of the California Current System (CCS). The present study examines CCS phytoplankton transcriptomic responses to added N substrates and links those responses to observed physiological responses.

The CCS is a highly dynamic environment where N availability varies with location and season and, together with other factors, controls phytoplankton community composition and productivity [13,16,19,20]. Along the coast, the upwelling of cold nutrient rich waters by Ekman transport leads to high NO_3_^-^ concentrations [13,21]. About 200 km offshore, the CCS system is bounded by the warm oligotrophic California Current (CC) which flows toward the equator [13]. The transition zone (TZ) between the coastal mesotrophic region and the oligotrophic CC is characterized by high-energy eddies and cross-stream jets that drive mesoscale variability in nutrients and phytoplankton productivity [22–24]. In the late spring and summer, upwelled nutrient-rich water travels offshore across the TZ and, along the way, becomes depleted of NO_3_^-^ due to biological activities and physical forces [13,14]. In the late summer and early fall, weaker nearshore upwelling followed by mixing of TZ and oligotrophic CC waters can make NO_3_^-^ scarce and limit phytoplankton primary production [13]. Moreover, iron (Fe) can be depleted faster than NO_3_^-^ leading to Fe limitation or N and Fe co-limitation for phytoplankton in the TZ [3,14].

In the coastal regions of the CCS, diatoms and other large photosynthetic eukaryotes drive primary production, whereas in the TZ, diverse and abundant photosynthetic picoeukaryotes and picocyanobacteria are major contributors [25–27]. Picocyanobacteria of the genera *Synechococcus* and *Prochlorococcus* are major contributors to primary production due to their abundance throughout the world ocean, including the CCS [28–30]. Both genera consist of several phylogenetic clades and ecological types (ecotypes) that occupy different niches based on temperature, nutrient, and light availability [7,30–38]. Multiple clades of *Synechococcus* and *Prochlorococcus* have been observed across the CCS from coastal to transition zones including novel clades or those that lack close reference genomes [39,40]. Generally, *Prochlorococcus* genomes are smaller and more GC-rich, and thus require less N compared to *Synechococcus* [34,41–44]. Under N limitation, some *Prochlorococcus* conserve N by using alternative transcription start sites to produce shorter proteins [45], whereas some *Synechococcus* make N available by degrading photosynthetic pigments [46]. The two genera vary in their abilities to utilize different N species. For example, while nearly all *Synechococcus* strains can assimilate NO_3_^-^ and NO_2_^-^, only some *Prochlorococcus* strains can [34,47]. Moreover, intrastrain variations in environmental sensing and nutrient assimilation capabilities have been observed [47–49] which makes it challenging to infer functional diversity from 16S rRNA gene sequences alone.

Deep sequencing approaches have become widely used in marine meta-omics and are effective for studying abundant community members, genes, and transcripts [50–54]. Alternatively, high-resolution microarrays have advantages for detecting rare community members and for differentiating among closely related strains [55–57]. The MicroTOOLs microarray targets functional genes in abundant and rare members of oligotrophic and coastal surface marine microbial communities, including picocyanobacteria (*Prochlorococcus* and *Synechococcus*), N_2_-fixing cyanobacteria, photosynthetic eukaryotic phytoplankton, as well as a small number of viruses [55]. Probes on the array can distinguish among closely related strains known from culture and environmental samples [55], and thus can elucidate the physiological state of individual microbial populations within a complex community [56].

In August 2014, we conducted experiments to determine the effects of different N substrates on phytoplankton communities at two stations, one in the North Pacific Ocean and one in the transition zone of the California Current (Stn. TZ), originally described in Shilova et al. [10]. Surface water samples were incubated with NO_3_^-^, ammonium (NH_4_^+^), urea, or filtered deep water (FDW) for 48 hours (T48). Two treatments had added Fe^3+^, either alone (“Fe”) or with a mix of N substrates (“N+Fe”), to determine the effects of Fe on the utilization of N substrates. After 48 hours, all treatments resulted in changes in phytoplankton cell abundances and photosynthetic activity at both locations, with differences between phytoplankton groups. *Prochlorococcus* had large increases in biomass in response to NH_4_^+^ and urea, while both eukaryotic phytoplankton and *Synechococcus* had highest biomass increases in response to FDW, and to N+Fe for *Synechococcus*. Moreover, distinct physiological responses were observed within sub-populations of *Prochlorococcus* and *Synechococcus*. In order to better understand the variable responses to N substrates among phytoplankton groups and sub-populations in the CCS transition zone, the present work used the MicroTOOLs microarray to examine transcriptomic changes that occurred 24 hours (T24) after the substrates were added. We hypothesized that transcript level changes at T24 would indicate which phytoplankton taxa were N-limited, and thus help explain changes in cell abundances for individual phytoplankton groups observed at T48. Furthermore, we hypothesized that the diversity in physiological responses within *Prochlorococcus* and *Synechococcus* would be evident in the transcriptomic responses measured at sub-population resolution.

## Materials and methods

### Experiment set up and processing

The experiment was conducted during the Nitrogen Effects on Marine MicroOrganisms cruise (NEMO; R/V New Horizon NH1417) between August 23–26, 2014 using surface seawater from Station 38 (33.502 °N, 129.37 °W) in the transition zone of the CCS, as originally described in Shilova et al. [10]. Briefly, 25 m water was collected using a towed swim fish and then gently pumped through 80 μm Nitex mesh to exclude large predators into a 40 L carboy, which allowed mixing of seawater before being distributed into 4 L polycarbonate bottles (Thermo Scientific^™^ Nalgene^™^, ThermoFisher Scientific, Waltham, MA, USA). Preparation of nutrient solutions and bottles and sampling of incubations were carried out under strict trace-metal clean conditions in a trace-metal clean laboratory van. All bottles were acid-washed and rinsed thoroughly with seawater at the site of the experiment. Triplicate bottles were treated with NO_3_^-^, NH_4_^+^, urea, Fe^3+^, Fe^3+^ with a mix of the three N substrates (“N+Fe”), or filtered 600 m water (filtered deep water; FDW). N substrates were added for a final concentration of 5 μM, while Fe was added for a final concentration of 2 nM. Deep water was collected from the same station where surface seawater was collected for the incubations (Station 38) at 15:30 on August 23, 2014 and filtered through Sterivex^™^ filters (Millipore, Billerica, MA, USA). Incubations took place in seawater cooled on-deck incubators, with shading to attenuate light to 35% incident levels. The set-up of the experiment took place on August 24^th^ with T0 samples collected for RNA at 1:00 and T24 samples collected pre-dawn on August 25th between 3:30 and 5:45. RNA samples were gently filtered using a peristaltic pump in the dark in under 15 minutes per sample, immediately flash frozen in liquid nitrogen and stored at -80° C until extraction. For T0, 4 L of seawater were filtered onto Sterivex^™^ filters (Millipore). For T24, 2 L of seawater were filtered onto 0.2 μm Supor membrane filters (Pall Corp., Ann Arbor, MI, U.S.A).

### RNA extraction and processing

Total RNA was extracted from the samples using the Ambion^®^ RiboPure RNA purification kit (ThermoFisher Scientific) with the addition of a bead-beating step during TRI Reagent extraction as described in Shilova et al. [55]. DNA was removed from the total RNA extracts in a solution using RNase-Free DNase Kit (Qiagen, Germantown, MD, USA), and RNA was purified again with RNA Clean & Concentrator^™^-25 (Zymo Research, Irvine, CA, USA) according to the manufacturers’ protocols. The RNA quality and quantity were evaluated using the Agilent BioAnalyzer RNA Nano Kit (Agilent, Santa Clara, CA, USA) and Qiagen Qubit. T0 and T24 RNA samples with an RNA Integrity Number greater than 9 were processed for microarray analyses. Three hundred ng of total RNA was used for synthesis of double-stranded cDNA using the TransPlex Whole Transcriptome Amplification 2 Kit (Sigma-Aldrich, St. Louis, MO, USA) following the manufacturer’s instructions. For amplification control, 0.5 μl of 1:100 dilution of the mRNA Ambion ERCC mix 1 (ThermoFisher Scientific) was spiked into each RNA sample prior to cDNA synthesis. Amplified transcriptome samples were purified with the GenElute PCR CleanUp Kit (Sigma Aldrich).

cDNA was labeled at the Roy J. Carver Center for Genomics (The University of Iowa, USA) using the Agilent SureTag DNA Labeling Kit (Cat# 5190–3400) following a protocol described in “Agilent Oligonucleotide ArrayBased CGH for Genomic DNA Analysis: Enzymatic Labeling for Blood, Cells, or Tissues (Version 7.3 March 2014)” (S1 File). Cy3-labeled cDNA was hybridized using a Gene Expression Hybridization Kit (Cat# 5188–5242) and following the protocol described in “One-Color Microarray-Based Gene Expression Analysis: Low Input Quick Amp Labeling (Version 6.7, September 2014)” (S1 File). Microarray platform GPL24371 Agilent-073391 MicroTOOLs_171K_oligo_v2.0 was used in this study. Microarrays were scanned at the Roy J. Carver Center for Genomics using an Agilent SureScan Microarray Scanner G2600D (Serial #: SG13134301) and the Agilent scanning protocol GE1_1200_Jun14 (Feature Extractor software version 11.5.1.1). The microarray data were submitted to The National Center for Biotechnology Information (NCBI) Gene Expression Omnibus (GEO) under accession GSE130464.

### Microarray analyses

Microarray analyses were done using the MicroTOOLs R package (https://www.jzehrlab.com/microtools) using the same approaches and parameters described in Robidart et al. [56] (robust multi-array averaging of probes and quantile normalization across samples), except that differentially expressed (DE) genes were identified based on a fold change significantly greater than 1.2 (Benjamini-Hochberg adjusted p-value <0.05; [58]). A total of 23 RNA samples were analyzed in this study, including three replicates for controls taken at T0 and T24 and three replicates for each treatment collected at T24, with the exception of the NH_4_^+^ treatment which had two replicates (the third NH_4_^+^ replicate did not pass microarray hybridization quality control and was excluded from the analysis). Multiple approaches were applied to examine responses at the phylogroup and individual strain levels and also due to variability among some treatment replicates (Table 1).

**Table 1:** Transcriptomic response analyses.

Analysis	Identifies	How applied	Ref.
NMDS	Metatranscriptome differences among samples (replicate consistency, treatments vs. controls).	Sample clusters compared within this study and to a NPSG study [56].	[59]
Single-gene DE	Individual genes that are significantly differentially expressed between two conditions.	FDW, NO_3_^-^, and urea treatments vs. controls at T24. Controls at T24 vs. at T0.	[60, 61]
EGSEA	Sets of genes that collectively are significantly differentially expressed between two conditions.	All treatments vs. controls at T24. Controls at T24 vs. at T0.	[62]
WGCNA	Modules of genes with highly correlated expression patterns across samples.	Gene expression profiles across T24 samples were correlated.	[63]

Transcription patterns were analyzed using three approaches. The single-gene DE analysis is the traditional approach for identifying differentially expressed genes. In the main text “DE” is used only for results from the single-gene analysis. NMDS—non-metric multidimensional scaling; DE—differentially expressed; EGSEA—Ensemble of Gene Set Enrichment Analyses; WGCNA—weighted correlation network analysis.

We used an Ensemble of Gene Set Enrichment Analyses (EGSEA; [62]) to identify sets of genes that collectively were significantly differentially expressed based on a consensus of twelve GSEA algorithms (Benjamini-Hochberg adjusted p-value < 0.01, unadjusted p-value calculated using Wilkinson’s method [64] to combine p-values from the GSEA algorithms). We defined each gene set to contain MicroTOOLs gene targets from a specific phylogroup and physiological response, e.g. genes from high-light (HL) adapted *Prochlorococcus* that typically increase during nitrogen limitation (Table 2). Each gene set was analyzed for differential expression in each treatment relative to controls at T24.

**Table 2:** Gene set definitions for EGSEA.

EGSEA gene set	Member genes
Fe stress	*fur*–ferric transcriptional regulator
	*isiA*–iron stress-induced chlorophyll-binding protein
	*isiP*–iron stress-induced protein
	*isiB*–flavodoxin
	*idiA*–iron (III) transporter
	*dpsA*–ferritin-like diiron-binding domain
N stress	*ntcA*—global N transcriptional regulator
	*urtA*—urea ABC transporter, substrate-binding protein
	*cynA*—cyanate ABC transporter, substrate-binding protein
	*amt*–ammonium transporter
	*glnA*—glutamine synthetase
	*ureA*—urease alpha subunit
	*nirA*–ferredoxin-nitrite reductase
	*nrtP*–nitrate transporter
P stress photosynthesis	*psiP*—phosphorus starvation inducible protein
	*pstS*—high-affinity phosphate-binding protein of phosphate ABC transporter
	*phoH*—phosphate stress inducible protein
	*mfs*—major facilitator superfamily transporter responding to P starvation in *Prochlorococcus* [65]
	*phoB*—Pho regulon transcriptional regulator
	*pstA*—permease protein of high-affinity phosphate transporter
	*pstB*—ATP-binding protein of high-affinity phosphate transporter
	*pstC*—permease protein of high-affinity phosphate transporter
	*psaB*—photosystem I P700 chlorophyll a apoprotein A10
	*psbA*— photosystem II PsbA protein (D1)
	*psbB*—photosystem II PsbB protein (CP47)
	*psaA*—photosystem I P700 chlorophyll a apoprotein A1
RuBisCO	*rbcL*—ribulose bisphosphate carboxylase large chain
light stress	*phrB*—DNA photolyase
	*nudix*—nudix hydrolase
	*pmm1359*—conserved light-responsive protein, identified in *Prochlorococcus* MED4

The EGSEA analysis identified collectively significant changes from the genes in each set. When EGSEA was applied to a phylogroup, all of the genes in each set had MicroTOOLs targets from multiple strains. When EGSEA was applied to a specific strain, all available genes for the strain were included. Some strains lacked targets for some genes, but usually each strain had multiple targets for every gene listed. Note that each stress gene set has member genes that all increase or decrease together when the stress changes.

The same volume of seawater was processed for metatranscriptomic analysis of each T24 sample, and neither the cell abundances for major phylogroups nor the microbial community composition, as measured with the 16S rRNA gene V4 sequencing, changed significantly by T24 [10]. Thus, the transcriptomic changes we report are not artifacts of community composition differences among treatments. However, the same amount of cDNA was hybridized to each microarray, therefore transcript level increases from one phylogroup could result in transcript level decreases for other phylogroups. We found that this artifact was too small to account for differentially expressed gene sets [described in S1 File and S1 Fig]. For each gene set *g* from phylogroup *p* analyzed for differential expression, the average fold change of member genes (*a*_*g*,*p*,*t*_) in treatment *t* vs. the control at T24 was compared to the average fold change in total transcripts from the phylogroup across replicates of the treatment (*a*_*tot*,*p*,*t*_ shown in S1 Fig for each phylogroup and treatment). Only differentially expressed gene sets with *a*_*g*,*p*,*t*_ / *a*_*tot*,*p*,*t*_ > 1 if an increase or *a*_*g*,*p*,*t*_ / *a*_*tot*,*p*,*t*_ < 1 if a decrease were further analyzed. Finally, we used weighted correlation network analysis (WGCNA; [63]) to identify genes with highly correlated expression patterns across all 20 of the T24 samples, mainly for genes that had strong patterns across taxa (e.g. *urtA*). Strains were considered to be present in a sample based on the detection of at least five of their target genes in that sample, or at least half of their targets if they had fewer than five targets (mainly photosynthetic eukaryotes).

## Results and discussion

### Transcripts were detected from oligotrophic and coastal microbial taxa

The surface microbial community at Stn. TZ 24 h after N addition was diverse and transcriptionally active. A total of 9760 genes had detectable transcripts in one or more of the 23 total samples (Materials and methods). Based on detected genes, samples had on average 575±24 distinct strains of the 924 included on the MicroTOOLs array. All taxa identified in control samples at T0 were also detected at T24 and represented major phylogroups found in the open ocean and coastal/transitional environments: picocyanobacteria (*Prochlorococcus* and *Synechococcus*), alpha-, gamma- and beta-proteobacteria, and eukaryotic phytoplankton including stramenopiles (e.g. diatoms) and haptophytes (Table 3).

**Table 3:** Detected strains.

Phylogroup	Strains detected in ≥ 1 sample	Strains detected in ≥ 20 samples	Total strains represented in MicroTOOLs
stramenopiles	147	132	157
alpha proteobacteria	95	70	115
*Synechococcus*	57	48	66
gamma proteobacteria	49	37	81
*Prochlorococcus*	43	27	48
beta proteobacteria	30	21	41
N_2_-fixing cyanobacteria	30	15	35
haptophytes	20	20	20
Euryarchaeota	17	12	36
dinoflagellates	8	6	9
other	188	129	316
Total (percentage of total in MicroTOOLs)	684 (74%)	517 (56%)	924

Many of the strains represented on the microarray were detected repeatedly across the 23 samples. For example, of 48 distinct *Prochlorococcus* strains represented on MicroTOOLs, 43 were detected in at least one sample and 27 were detected in at least 20 samples. Strains are categorized into major phylogenetic groups.

Some of the highest transcript relative abundances, hereafter referred to as “transcript levels,” were detected from the dominant picocyanobacteria that Shilova et al. [10] identified using 16S rRNA gene V4 sequencing and oligotyping analysis, including high-light adapted (HL) *Prochlorococcus* strains MED4 and MIT9515 (Table 4, S1 Table). However, other strains detected in the present study were rare or not observed by Shilova et al. [10], and had even higher transcript levels than some of the “dominant” strains from the same ecotype. For example, HL *Prochlorococcus* strain MIT9301 comprised just 0.33% of the total *Prochlorococcus* 16S rRNA gene sequences, but in 18 of the 23 samples it had a higher total transcript intensity (normalized to detected genes) than MIT9515.

**Table 4:** Detected picocyanobacteria.

Genus	Strain	Clade	Clade environment	16S-rRNA rel. abund.^†^ (%)	Transcript rel. abund. range	MicroTOOLs gene targets detected and (%)
*Prochlorococcus*	MED4	HLI	open ocean, temperate lat. [33]	79.9	9.1–10.3	283 (65)
	MIT9515	HLI	open ocean, temperate lat. [33]	12.1	9.1–10.1	200 (67)
	MIT9301	HLII	open ocean, subtropical and tropical lat. [33]	0.3	9.9–10.4	368 (57)
	NATL1A	LLI	open ocean, deep euphotic zone [32]	1.6	8.3–9.0	47 (67)
	MIT9313	LLIV	open ocean, deep euphotic zone [32,66]	-	14.9–16.1	38 (79)
*Synechococcus*	CC9605	II	open ocean and coastal, tropical and sub-tropical lat. [33,38]	50.2	8.0–8.7	147 (85)
	CC9902	IV	coastal, high lat., cold [38]	39.9	6.7–7.0	139 (77)
	CC9311	I	coastal, high lat., cold [33,38]	-	6.8–7.2	177 (79)
	JA-2-3b’a(2–13)	none	hot spring [67]	-	21.8–28.3	7 (88)
	RS9917	VIII	coastal, hypersaline [68]	-	9.0–9.8	180 (91)
	WH7805	VI	coastal / transitional [33]	-	9.7–10.7	124 (78)
	WH5701	5.2	estuary [29]	-	13.2–14.6	377 (94)
	WH8102	III	open ocean, low-nutrient [33,38]	-	7.6–8.3	397 (87)

*Prochlorococcus* clades are adapted to high-light (HL) or low-light (LL).

^†^: relative abundances based on 16S rRNA gene V4 sequencing in [10]

-: the strain was not detected by 16S rRNA gene V4 sequencing in [10]

In the T0 controls, some picocyanobacteria that were not detected by 16S rRNA gene sequencing had higher transcript levels than dominant strains. The 16S rRNA gene copy relative abundances are percentages for each strain relative to all 16S rRNA gene copies detected for the genus as identified earlier [10]. Transcript relative abundance range is the strain’s proportion of total detected transcripts, divided by the strain’s total target genes on MicroTOOLs, scaled to 1E5. The Clade environment column characterizes the ocean region and latitude where the clade is often present. For each strain, the last column indicates the number of detected target genes in T0 controls and the percentage relative to MicroTOOLs target genes for the strain in parentheses.

Similar to *Prochlorococcus*, we detected transcripts from two dominant *Synechococcus* strains previously identified at Stn. TZ using 16S rRNA gene sequencing [10], CC9605 (clade II) and CC9902 (clade IV), as well as other clades; however, transcripts from CC9902 were rare (Table 4, S1 Table). Given the rarity of *Synechococcus* at Stn. TZ (relative abundance ~0.8% based on 16S rRNA gene abundances, or 3.9±0.7 × 10^3^ cells mL^-1^; [10]), the diversity of detected *Synechococcus* strains demonstrated the sensitivity of MicroTOOLs for studying rare but transcriptionally active community members.

Photosynthetic eukaryotes (PE), including stramenopiles (mainly bacillarophytes [diatoms]), haptophytes, and other groups (e.g. ochrophytes, chlororphytes, and cryptophytes) are almost exclusively represented on MicroTOOLs by *rbcL* genes (353 targets total) which encode the large subunit of RuBisCO. Transcripts were detected for 296 PE *rbcL* targets, from all represented PE phylogroups. Transcripts from other organisms were also detected including the heterotrophic bacteria *Pelagibacter* SAR11 (primarily *Pelagibacter ubique* spp.), HTCC7211, HTCC1062, HTCC1002, and also from viruses (cyanophages) (Table 3; S1 File; S1 Table).

Altogether, we observed numerous strains of PE, *Prochlorococcus* (43 types), and *Synechococcus* (57 types), based on transcripts detected from multiple gene targets for the picocyanobacteria (Materials and methods). The numbers of *Prochlorococcus* and *Synechococcus* strains were higher than reported by Shilova et al. [10] using 16S rRNA gene oligotyping (11 *Prochlorococcus* and 31 *Synechococcus*). The complex ensemble of detected open ocean and coastal strains of phytoplankton may reflect mesoscale processes in the CCS, such as the circulation of baroclinic jets and eddies [23], as well as late summer physical forces that mix open ocean and transition zone waters [69]. However, the stability of and dynamics within such mixed communities are unknown. Physiologically, they respond differently to nutrients than open ocean communities [10]. The results that follow examine transcriptional changes that underly the physiological responses of this mixed phytoplankton community to nutrient availability.

### Nutrient additions resulted in distinct transcription patterns

**Fig 1:**
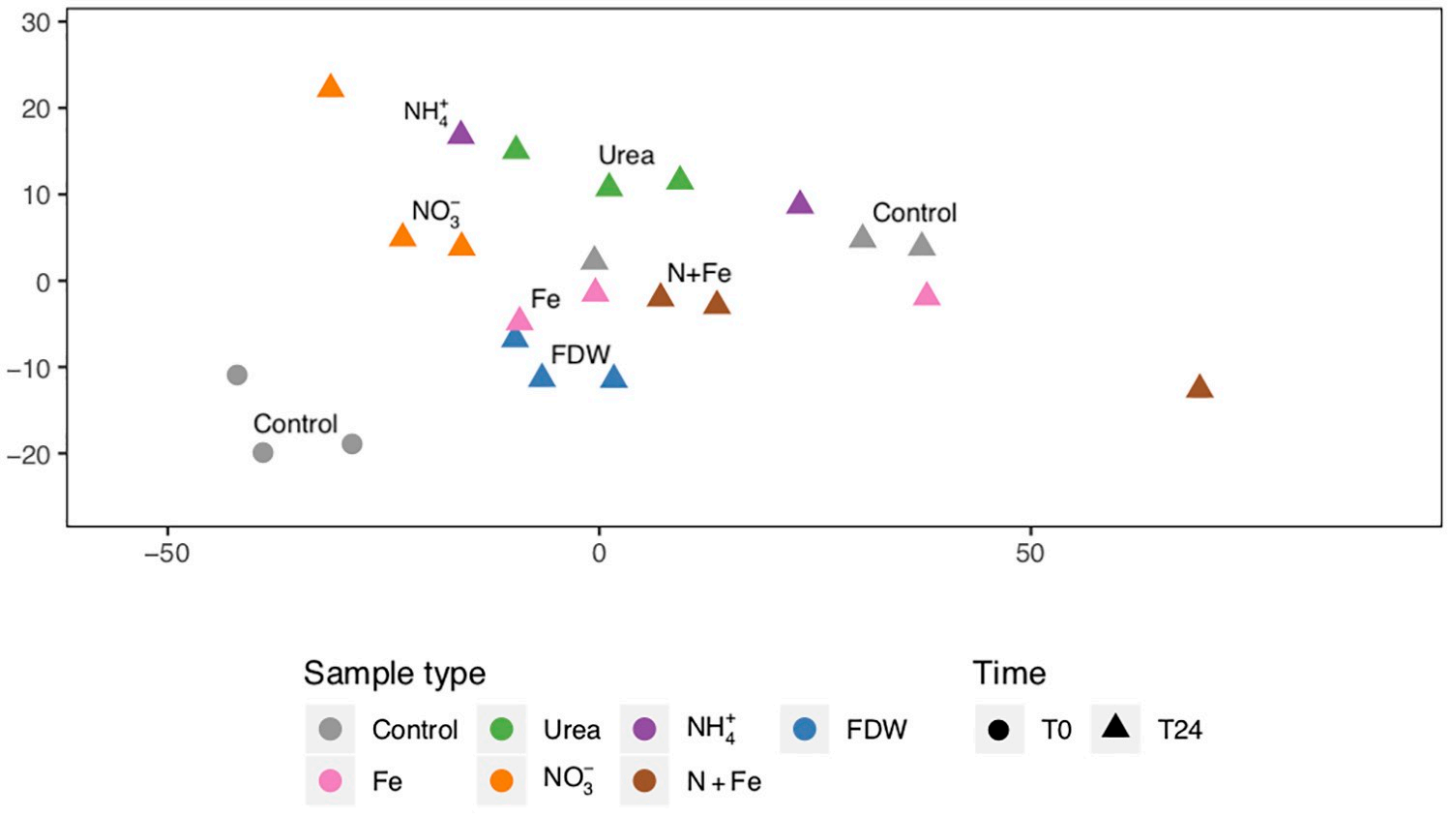
NMDS of the metatranscriptomes. Non-metric multidimensional scaling (NMDS) was used to analyze the log_2_ transcript levels of the 9760 total detected genes in the 23 samples. Euclidean distances between samples were analyzed. Stress was 0.06.

The transcription pattern for each sample, its metatranscriptome, was defined by the transcript relative abundances for the 9760 total detected genes. Metatranscriptomic changes that occurred within 24 hours of nutrient additions (Fig 1 and S2 Fig) helped explain longer-term physiological changes observed at T48 by Shilova et al. [10]. For example, the urea and NH_4_^+^ treatments at T24 clustered further from the T0 controls than most other treatments (median pairwise Euclidean distances to T0 controls were 50.6 and 54.3 for the urea and NH_4_^+^ treatments, respectively, versus 42.5 and 47.1 for the NO_3_^-^ and the FDW treatments, respectively, S2 Table) and resulted in the largest increases by T48 in Chl *a* concentrations, primary productivity rates, and cell densities of the most abundant group of phytoplankton, *Prochlorococcus* (>2-fold) [10]. In comparison to the urea and NH_4_^+^ treatments, the NO_3_^-^ and FDW treatments resulted in metatranscriptomes that were more similar to the T0 controls (Fig 1) and by T48 had smaller (but significant) increases in Chl *a* concentrations, primary productivity rates, and *Prochlorococcus* cell densities [10]. Unlike *Prochlorococcus*, *Synechococcus* and PE cell densities did not strikingly increase in the urea and NH_4_^+^ treatments in comparison to the NO_3_^-^ and FDW treatments [10].

The MicroTOOLs design includes far more probes that target *Prochlorococcus* and *Synechococcus* than PE and heterotrophic bacteria. Thus, the similarity of NO_3_^-^ and FDW metatranscriptomes to T0 metatranscriptomes, together with the significant increases in Chl *a* concentrations and primary productivity rates, suggest that other bacteria and eukaryotes had advantages over *Prochlorococcus* for responding to higher concentrations of NO_3_^-^ and other nutrients in FDW [70,71]. Furthermore, the responses to FDW were similar to those in another NPSG study that used MicroTOOLs (see NMDS in [56]; *in situ* samples in that study are analogous to controls at T0 here). Both studies have captured metatranscriptomic changes from surface microbial communities responding to influxes of nutrients, such as those expected as a result of anticyclonic eddies.

Fe was likely not a limiting nutrient for the microbial community. Metatranscriptomes from treatments with Fe or N+Fe were more similar to controls at T24 and also more variable compared to treatments with only N substrates (except for NH_4_^+^; Fig 1). This suggests that the community, as measured with the microarray, responded more to N than to Fe additions. Consequently, cell abundances were not significantly higher in N+Fe treatments compared to NO_3_^-^ alone [10].

In a separate NMDS analysis, the metatranscriptomes from this CCS study clustered more tightly than metatranscriptomes from a NPSG study that investigated the effects of deep water mixing on the surface microbial community ([56]; S3 Fig, mean within-study Euclidean distances, before NMDS, were 45.5 for CCS versus 139.6 for NPSG). This suggests that the CCS surface community metatranscriptomes were less perturbed over a 24 h period by the addition of nutrients than were the NPSG metatranscriptomes by the addition of deep water (S1 File). The small relative abundance changes and lack of changes in photosynthetic efficiency by T24 in the CCS samples relative to NPSG samples from the same study [10] support this conclusion.

### *Prochlorococcus* had similar responses to NH_4_^+^ and urea

**Fig 2:**
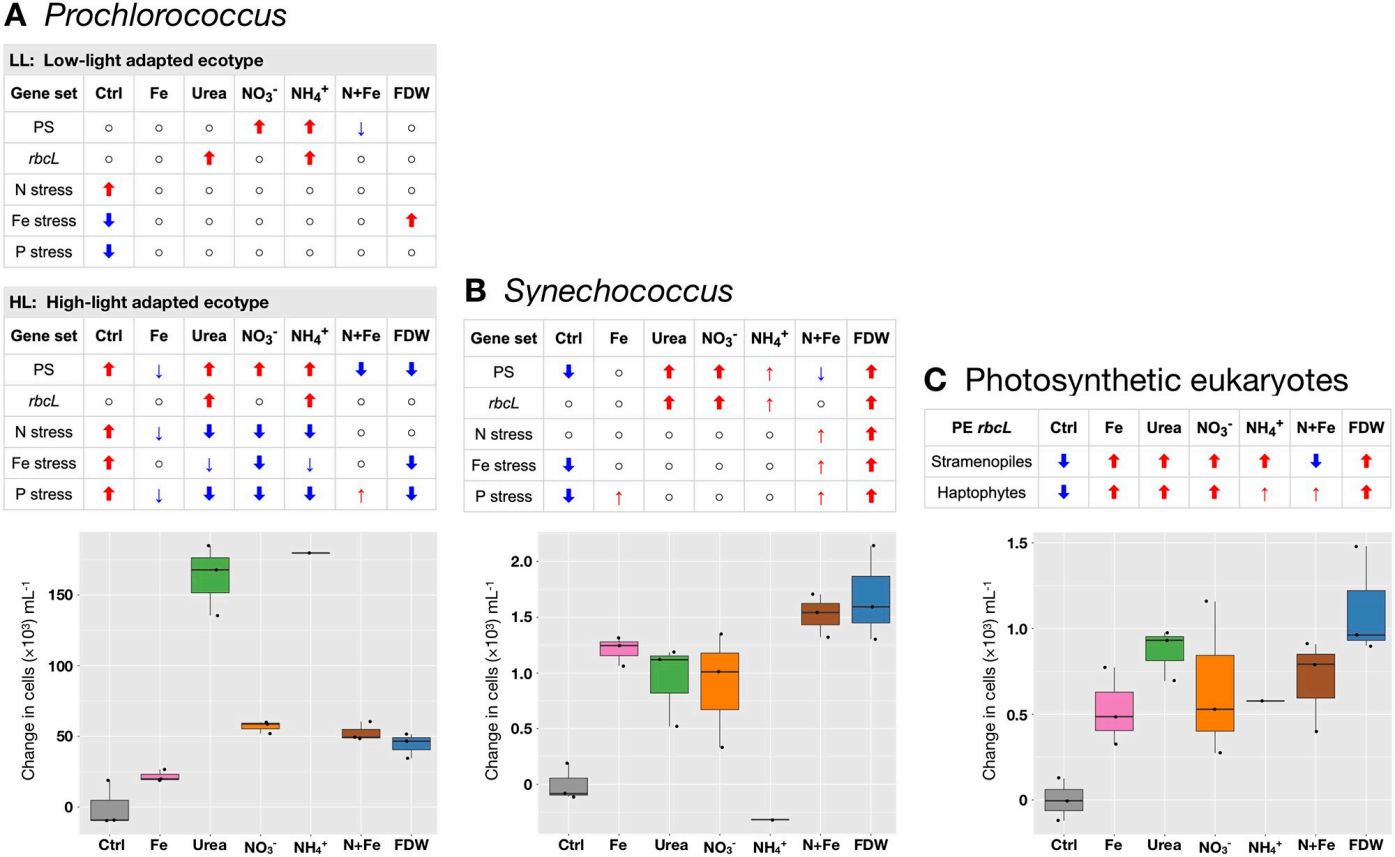
Differentially expressed gene sets (T24) and cell abundance changes (T48). For major phylogroups *Prochlorococcus* (A), *Synechococcus* (B), and photosynthetic eukaryotes (C), significantly differentially expressed gene sets (EGSEA) at T24 are shown with corresponding cell abundance changes by T48 (relative to controls at T48; [10]) for each treatment. In the EGSEA results, red and blue arrows indicate significant (p < 0.01) increases and decreases, respectively, for the indicated gene sets (Materials and methods, Table 2). Thick arrows additionally mean that the average fold change for genes in the set was >1.2×. Cell abundance changes for *Prochlorococcus* are for HL and LL ecotypes combined. ◦: change was not significant.

**Fig 3:**
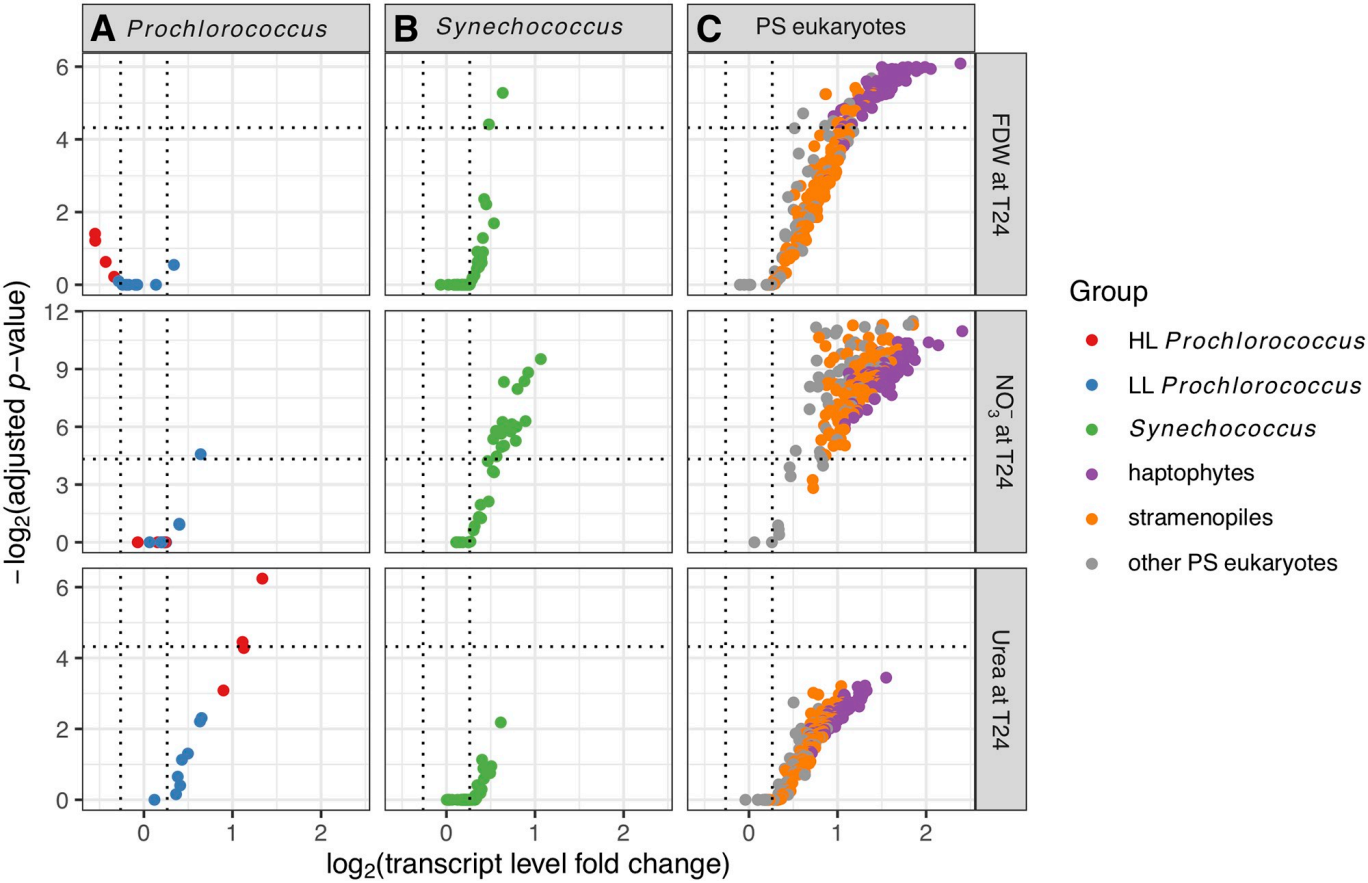
Phytoplankton *rbcL* gene responses. Transcript level changes for *rbcL* genes for HL and LL *Prochlorococcus* (A), *Synechococcus* (B), and photosynthetic eukaryotes (C) in the FDW, NO_3_^-^, and urea treatments. In these treatments, the single-gene differential expression (DE) analysis (Materials and methods) identified genes that changed significantly in comparison to controls at T24. DE genes are above the horizontal dotted lines and have significant fold changes >1.2×, indicated by the vertical dotted lines. Note that *y* axes differ across treatments.

**Fig 4:**
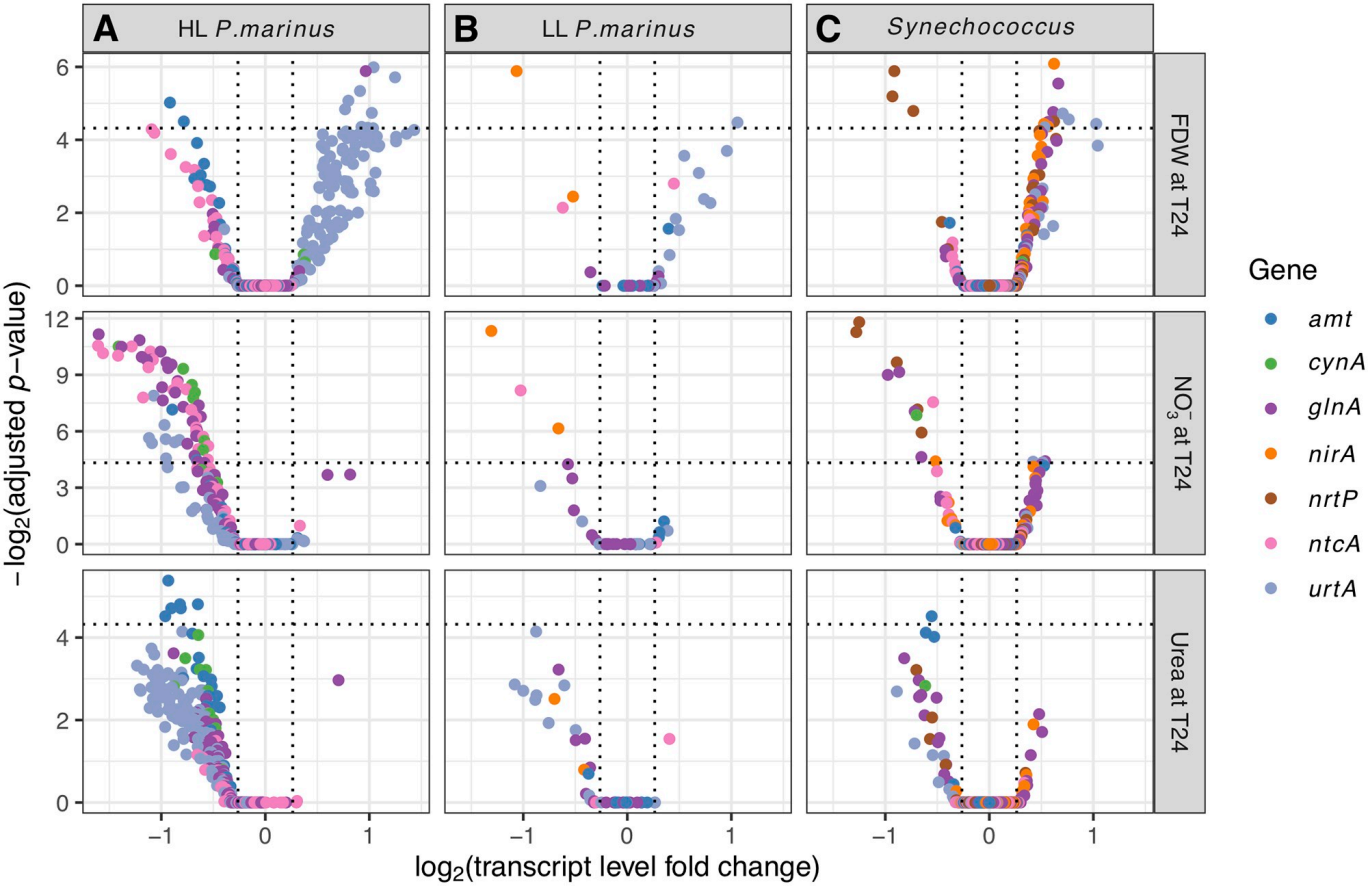
Picocyanobacteria N stress gene responses. Responses from N stress genes for HL (A) and LL *Prochlorococcus* (B), and *Synechococcus* (C) in the FDW, NO_3_^-^, and urea treatments. These genes are generally up- or down-regulated depending on whether the cell is N-limited or replete. Conventions are as in Fig 3.

**Fig 5:**
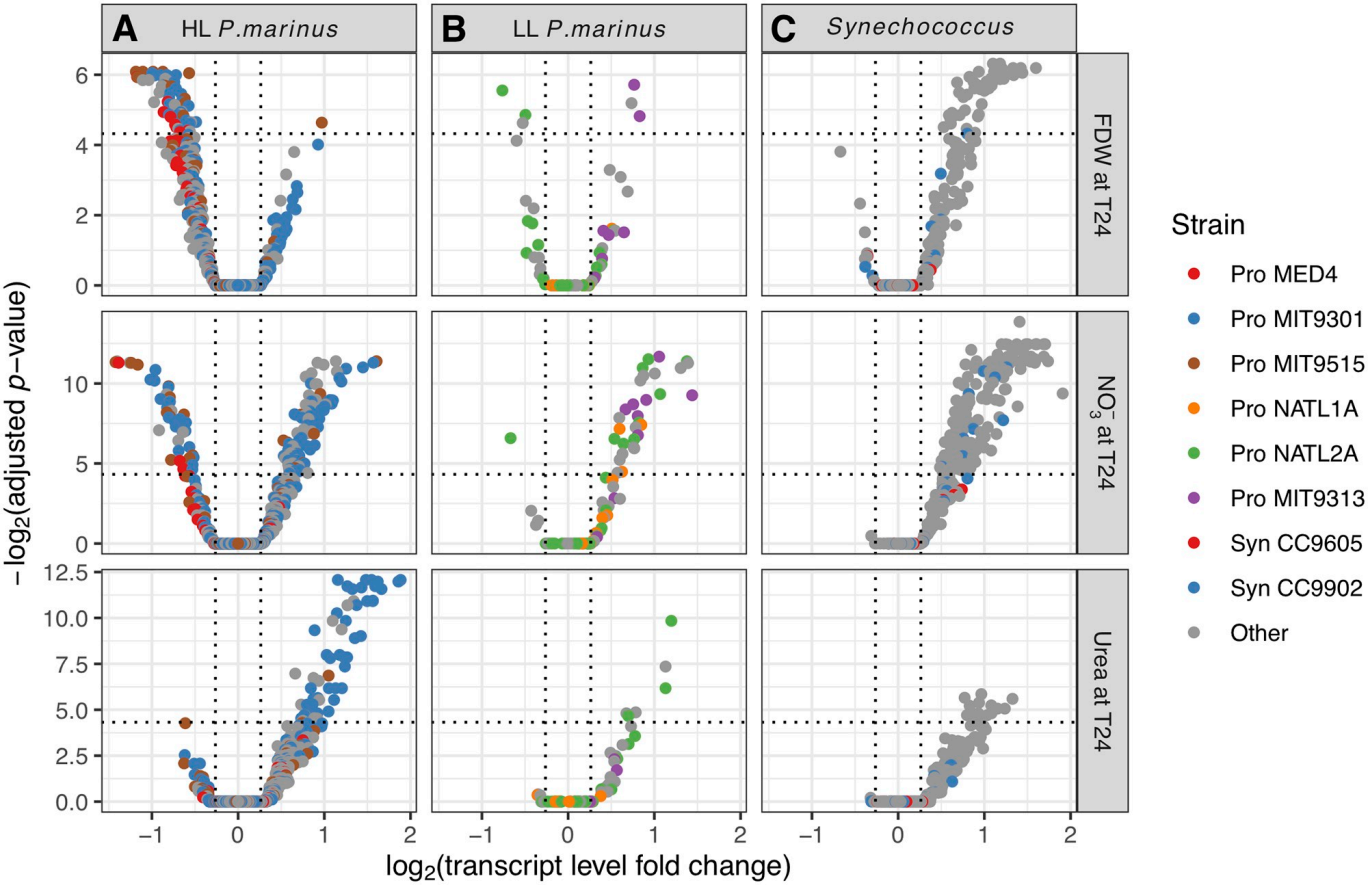
Picocyanobacteria PS gene responses. Responses from photosynthesis genes for HL (A) and LL *Prochlorococcus* (B), and *Synechococcus* (C) in the FDW, NO_3_^-^, and urea treatments. Conventions are as in Fig 3.

*Prochlorococcus* showed signs of alleviation of N stress 24 h after the NH_4_^+^ or urea addition as evidenced by transcript decreases for N stress associated genes and increases for photosynthesis (PS) and carbon fixation genes (*rbcL*; Figs 2A, 3A, 4A, 4B, 5A and 5B). The Ensemble of Gene Set Enrichment Analyses (EGSEA) showed that N stress gene transcripts decreased for HL *Prochlorococcus* strains overall (Fig 2A), as well as for the dominant strains MED4 and MIT9515 (S1 Table). Although N stress genes across LL strains did not change significantly in the NH_4_^+^ or urea treatments, their carbon fixation genes were up-regulated in both treatments. PS genes were up-regulated in the NH_4_^+^ treatment across the low-light adapted (LL) *Prochlorococcus* strains (Fig 2A) and for multiple LL strains in the urea treatment (NATL2A, MIT9211, CCMP1375; S1 Table).

When cyanobacteria experience low NH_4_^+^ conditions, the global nitrogen transcriptional regulator NtcA responds to high intracellular C-N ratios by activating transcription of genes associated with N acquisition and/or N stress, including *ntcA* itself [72–75]. Although we did not observe significantly differentially expressed (DE) *ntcA* from *Prochlorococcus* in the NH_4_^+^ or urea treatments, transcripts from most N transport and metabolism genes decreased (non-DE) in the urea treatments (Fig 4A and 4B). These genes included the ammonium transporter *amt*, carbamoyl-phosphate synthase *carA*, and glutamine synthetase *glnA*. Moreover, EGSEA indicated that collectively all of these N transport and metabolism genes (Materials and methods Table 2) had significant decreases for HL *Prochlorococcus* in both treatments, as described above (Fig 2A).

Analysis of relative transcript levels for *rbcL* versus *ntcA* indicated that treatments with NH_4_^+^ or urea substrates provided enough N to shift the internal C-N balance in *Prochlorococcus* MED4 within 24 hours (S1 File, S4 Fig). The up-regulation of PS and *rbcL* genes and down-regulation of N stress genes in response to urea or NH_4_^+^ are consistent with the highest increases of *Prochlorococcus* cell abundances observed in these treatments at T48 [10] (Fig 2A) and further support the ability of natural populations of *Prochlorococcus* to assimilate either substrate [12,43,76].

### Responses to nitrate varied within coexisting *Prochlorococcus* populations

Nitrate assimilation by both HL and LL ecotypes of *Prochlorococcus* is more widespread than was originally believed based on cultured strains [11,12,77,78]. At Stn. TZ transcriptomic responses from PS and N-stress genes to added NO_3_^-^ indicated a mix of NO_3_^-^ assimilation capabilities among *Prochlorococcus*, even within closely related populations. In the NO_3_^-^ treatment, PS transcript levels decreased for the most abundant HL *Prochlorococcus*, similar to MED4 (Fig 5A; S1 Table), suggesting that it did not utilize NO_3_^-^ which is consistent with non-growth of MED4 on NO_3_^-^ in culture [78]. However, 8 of the 11 detected MED4-like *ntcA* genes had decreased transcript levels suggesting the presence of MED4-like subpopulations at Stn. TZ that utilized NO_3_^-^ or reduced N available from other cells utilizing NO_3_^-^ (Fig 4A; S1 Table). Subpopulations with different abilities to utilize NO_3_^-^ were also suspected for the second most abundant *Prochlorococcus* population, MIT9515-like, as well as for the rare *Prochlorococcus* MIT9301-like population, based on mixed PS responses within each strain (Fig 5A; S1 Table). The FDW treatment had a similar NO_3_^-^ concentration as the treatment with NO_3_^-^ alone and also resulted in diverse responses from *Prochlorococcus* PS and N-stress genes (Figs 4A, 4B, 5A and 5B; S1 Table).

Altogether, the results showed that specific *Prochlorococcus* subpopulations in the CCS were utilizing NO_3_^-^ and that utilization varied within closely related populations. A mixed population might explain why *Prochlorococcus* cell abundance increases by T48 were less (but significant) in the treatments that added NO_3_^-^ than in the treatments that added urea or NH_4_^+^ [10,43] (Fig 2A). It is also possible that the *Prochlorococcus* subpopulations that responded positively to NO_3_^-^ were either minor members of the *Prochlorococcus* community and/or growing more slowly on NO_3_^-^ than on reduced nitrogen sources [78]. Mixed populations of *Prochlorococcus* strains with regards to their NO_3_^-^ utilization capabilities have been observed at Pacific and Atlantic sampling sites [79] and specifically within MED4-like subpopulations in the CCS [80]. The high variability in NO_3_^-^ uptake rates was also reported recently among cells within phytoplankton groups including *Prochlorococcus* at both CCS and NPSG locations [12] suggesting that intra-population heterogeneity in NO_3_^-^ utilization is likely widespread.

### *Synechococcus* was N-limited but had dissimilar response patterns to *Prochlorococcus*

Transcriptomic changes indicated that *Synechococcus* likely assimilated the added N substrates. For each of the treatments with N substrates alone (urea, NO_3_^-^, or NH_4_^+^) or with FDW, transcript levels increased from *rbcL* and PS genes from *Synechococcus* strains collectively (Fig 2B), as well as from individual strains (Figs 3B and 5C; S1 Table). For example, both the abundant *Synechococcus* CC9605 and CC9902 and the rare CC9311 had increased transcript levels of *rbcL* (all DE) and PS genes (all DE except for CC9605) in the NO_3_^-^ treatment. These results were in line with the observed *Synechococcus* abundance increases by 48h in all treatments except for the NH_4_^+^ treatment (Fig 2B; [10]), and consistent with N assimilation by *Synechococcus* in culture studies [81–83].

In contrast to the strong up-regulation observed for *rbcL* and PS genes across *Synechococcus*, a mixed response was observed for N stress genes though mainly from non-dominant strains. In the NO_3_^-^ treatment, transcript levels decreased for one *ntcA* gene from strain BL107, and for the nitrate/nitrite permease (*nrtP*) gene from strains WH7805 and RCC307 (Fig 4C). In the urea treatment, *amt* transcript levels decreased for RS9917. However, the EGSEA analysis did not show a collective decrease for *Synechococcus* N stress genes in response to N substrates alone, unlike for HL *Prochlorococcus* (Fig 2A and 2B).

Despite the low abundance of *Synechococcus*, its *ntcA* transcript levels were on average 3× higher than those of (abundant) *Prochlorococcus* (0.45±0.02 vs. 0.16±0.01% of total transcripts from all taxa, respectively). The high *ntcA* transcript levels, low variability in *rbcL* to *ntcA* transcript levels (S1 File, S4 Fig), and lack of changes for N stress genes from *Synechococcus* in N treatments might be due to its different strategy for adapting to low reduced N environments in comparison to *Prochlorococcus*. Some *Synechococcus* strains, such as open ocean strain WH8103, maintain the ability to transport and utilize oxidized N forms even if NH_4_^+^ is present [83,84]. Moreover, *Synechococcus* WH7803 expresses *ntcA* at a low level in the presence of NH_4_^+^ [85]. Maintaining the ability to utilize oxidized N forms might be an energetically favorable strategy for *Synechococcus* in a high light and low reduced N environment such as the open ocean.

The lack of an overall response from *Synechococcus* N stress genes might also indicate that some strains were N and Fe co-limited as supported by physiological results [10]. For example, cell abundance increases for a strain of KORDI-100 were nearly twice as much in response to the N+Fe treatment (significant) compared to the Fe addition alone (not significant) [10]. In contrast, treatments with N+Fe and Fe alone resulted in similar cell abundance increases for dominant strains within CC9605 and CC9902 [10]. Transcriptional changes from Fe stress genes differed by strain (S5 Fig; S1 Table), suggesting strain-specific Fe requirements in *Synechococcus* and in line with our hypothesis of N and Fe co-limitation. For example, in response to FDW or NO_3_^-^, transcript levels from ferric transcriptional regulator *fur* genes decreased for CC9902 (DE for one *fur* target), suggesting reduced Fe stress, while *fur* transcript levels mainly increased for other strains (non-DE; S5 Fig; S1 Table). The differences in Fe requirements [34,86,87] may dictate which *Synechococcus* strains dominate CCS waters under Fe limitation [6,88]. Consistent with strain-specific Fe requirements, the collective responses from *Synechococcus* to Fe (alone or with N) was indistinct. In the Fe treatment, genes associated with Fe stress as a whole did not decrease, nor did *rbcL* or PS genes increase as was observed in treatments that added N alone, or FDW which produced the largest *Synechococcus* cell increases (Fig 2B). Thus, the physiological and transcriptional changes together suggest that Fe benefited some strains but that N was the primary limiting nutrient for the majority of *Synechococcus* strains.

In summary, the transcriptomic results suggest that most *Synechococcus spp*. at Stn. TZ were N-limited. The addition of N substrates led to increases in PS and *rbcL* transcripts, and the results support findings that natural populations of *Synechococcus* utilize NH_4_^+^, NO_3_^-^, and urea [12]. However, there was a lack of response from *Synechococcus* N stress genes, in comparison to *Prochlorococcus*, which may have resulted from differences in Fe requirements or N metabolic strategies. The more abundant community members, such as *Prochlorococcus* and high nucleic acid (HNA) heterotrophs, likely outcompeted *Synechococcus* for N substrates, especially in urea and NH_4_^+^, partly due to diffusion advantages afforded by their larger surface to volume ratios [89–91]. These factors likely prevented substantial growth of *Synechococcus* in comparison to *Prochlorococcus* and HNA in N treatments by T48 [10].

### Upregulation of urea uptake genes by picocyanobacteria in FDW

In the FDW treatment, the up-regulation of picocyanobacterial urea transporter genes *urtA*, but not other N genes under NtcA regulation, suggested additional regulatory controls for *urtA* besides classic NtcA regulation. Specifically, transcripts for nearly all *Prochlorococcus urtA* genes increased while transcripts for other genes associated with N stress and under NtcA control, such as *ntcA* and *amt*, decreased (Figs 2A, 4A and 4B; S1 Table). *Synechococcus urtA* also was up-regulated in the FDW treatment (Fig 4C), and WGCNA analysis indicated that *urtA* transcription patterns were similar across picocyanobacteria but distinct from nearly all other detected N stress genes (S1 File). Similar to other N stress genes that are under NtcA control, known *urtA* promoters in *Prochlorococcus* and *Synechococcus* have a putative binding site for NtcA [92], and *urtA* is up-regulated under N limitation in *Prochlorococcus* HL and LL strains [93]. However, it seems unlikely that the increases of picocyanobacterial *urtA* transcripts observed in the FDW treatment were due to classic NtcA control. Diel expression of cyanobacteria *urtA* [94] also seems an unlikely explanation because *urtA* decreases would have been expected in our pre-dawn samples (S1 File).

Possibly, the *urtA* transcript level increases were specific to the availability of urea, which would benefit picocyanobacteria as both a N and C source [95]. A recent NanoSIMS study showed that both *Prochlorococcus* and *Synechococcus* assimilated urea during short (~5 h) incubations [12]. Urea might have been generated by other community members in the FDW treatment. Heterotrophic bacteria have been shown to produce substantial amounts of urea in the Southern California Bight [96], and in Shilova et al. [10], heterotrophic bacteria cell density (HNA cells mL^-1^) more than doubled (by T48) in response to FDW. Eukaryotic phytoplankton, which responded rapidly to FDW in our study, might also have produced urea [97–99]. The delay between FDW addition and urea production might explain why picocyanobacteria *urtA* was up-regulated at T24 in FDW but down-regulated at T24 in the urea treatment. If *urtA* is a fast responding gene, 24 hours might have been too late to detect its up-regulation in the urea addition treatment.

Alternatively, chemical or biological interactions after mixing of surface waters with deep waters could have triggered the up-regulation of *Prochlorococcus* and *Synechococcus urtA*. Robidart et al. [56] also observed *urtA* increases from *Prochlorococcus* 24 h after the addition of sub-euphotic zone (130 m) FDW to the surface community in the NPSG. However, other studies did not observe up-regulation of *urtA* in response to the addition of high-molecular weight dissolved organic matter [52] or the addition of deep seawater from 700 m to surface NPSG microbial communities [100]. Thus, the *urtA* response observed in the present study and [56] might be specific to conditions or picocyanobacterial populations. Future culture-based and *in situ* experiments will help determine whether urea transporter responses reflect the regulation of one or multiple copies of *urtA*, by NtcA and possibly other transcription factors [34].

### Photosynthetic eukaryote RuBisCO transcript levels increased in N treatments

All nutrient additions elicited *rbcL* transcriptional responses from photosynthetic eukaryotes (PE) and PE cell abundance increases by T48. The PE *rbcL* response to NO_3_^-^ was robust, with significant (DE) transcript level increases for 283 of the 296 detected PE *rbcL* targets, including all detected haptophyte targets and most detected stramenopile targets (mainly for diatoms on MicroTOOLs; Fig 3C). Transcript levels also increased for PE nitrate reductase (*Nr*) targets in the NO_3_^-^ treatment (3 DE for diatoms from *Amphora*, *Phaeodactylum*, and an undetermined genus; S1 Table), consistent with the activation of *Nr* transcription in the presence of nitrate in PE [101,102]. Diatoms use nitrate opportunistically [70,71] rather than as a preferred N form [12], and haptophytes can utilize nitrate as well [103,104]. Thus, the *rbcL* and *Nr* transcript increases may indicate that both groups utilized the added NO_3_^-^ (~5 μmol L^-1^) because other N forms were scarce at Stn. TZ ([NH_4_^+^] = 58±3 nmol L^-1^ and [NO_3_^-^ + NO_2_^-^] = 2.6 ± 0.4 nmol L^-1^; [10]). Although the FDW and the NO_3_^-^ treatments had approximately the same NO_3_^-^ concentration, FDW resulted in fewer DE increases for *rbcL* and *Nr* targets for both haptophytes and stramenopiles (88 *rbcL*, and 1 *Nr* from *Phaeodactylum tricornutum*; Fig 3C; S1 Table). The urea treatment resulted in no DE genes from PE (however, 294 *rbcL* genes had non-DE transcript increases).

Like the single-gene DE analysis described above, the EGSEA analysis found significant *rbcL* transcript level increases for PE in the NO_3_^-^ and FDW treatments, but it also identified large (>1.2×), significant increases in the urea, NH_4_^+^, and Fe treatments (Fig 2C). The *rbcL* increases were followed by significant PE cell abundance increases at T48 in all treatments with the highest increase in FDW (Fig 2C) [10].

The concentration of Fe at Stn. TZ was below the level of detection (0.058 nmol L^-1^; [10]). The Fe treatment added Fe^3+^ to a final concentration of 2 nmol L^-1^ [10], whereas [6] estimated that Fe concentrations >0.5 nmol L^-1^ are enough for even the largest coastal CCS diatoms to grow if other nutrients are not limiting. The Fe treatment resulted in *rbcL* transcript increases from stramenopiles and haptophytes and modest PE abundance increases by T48 [10] (Fig 2C). Thus, for PE the Fe addition was sufficient for growth and the low initial concentrations of NO_3_^-^ and NH_4_^+^ (above) were not limiting. It was therefore surprising that the N+Fe treatment, which also added Fe^3+^ to 2 nmol L^-1^, resulted in *rbcL* transcript decreases from stramenopiles (Fig 2C). One potential explanation is that the added N substrates were used by N and Fe co-limited microorganisms which then outcompeted those detected by MicroTOOLs. Competition might also explain why haptophyte *rbcL* transcript increases were smaller in the N+Fe treatment (Fig 2C). Moreover, the differences in *rbcL* responses between stramenopiles and haptophytes to N+Fe indicate that haptophytes have lower Fe requirements than stramenopiles at Stn. TZ [105]; Fig 2C).

## Conclusions

This study and the previous by Shilova et al. [10] together provide a high-resolution snapshot of phytoplankton biodiversity in the CCS transition zone along three dimensions: taxonomic, transcriptional, and functional. The differential transcriptomic and physiological responses to N forms revealed in these two studies indicated that N was the primary limiting nutrient, but the responses differed with N substrate among *Prochlorococcus*, *Synechococcus*, and PE. Transcriptomes of some *Synechococcus* and PE populations indicated possible N and Fe co-limitation, in line with highest cell abundance increases after the addition of N+Fe for *Synechococcus* or FDW for both phylogroups [10]. Diverse transcriptomic responses were observed among closely related strains and sub-populations within *Prochlorococcus* and *Synechococcus*, indicative of different N assimilation capabilities and/or degrees of N limitation. For example, we observed heterogeneous populations of *Prochlorococcus* in their capacity to utilize NO_3_^-^, supporting previous single-cell nutrient uptake rate findings [12]. In the treatment with FDW, the unexpected increase of picocyanobacterial *urtA* while other N stress genes decreased highlights our incomplete understanding of urea utilization by marine microorganisms. The differences among natural phytoplankton populations in transcriptional and physiological responses were likely due to many factors including genetics, competition, and prior environmental conditions. Gene transcripts were detected from a mixed community of open ocean, transitional, and coastal strains reflecting the dynamic but poorly understood physical-biological interactions in the CCS transition zone. In fact, heterogeneity in responses within this mixed CCS microbial community might be a reason for the less pronounced whole transcriptome responses to added N observed in this study in comparison to the responses of the oligotrophic gyre community observed in a previous MicroTOOLs study [56]. Future studies along the dimensions of biodiversity at multiple locations and seasons will provide a more complete picture of how available N forms impact phytoplankton communities in this dynamic and productive part of the Pacific Ocean.

## Supporting information

S1 File Additional details on methods and results. (DOCX)

S1 Table Detected strains and genes. Spreadsheet containing normalized log_2_ transcript levels for all 9760 detected genes and whether they were DE, as well as their taxonomic and functional annotation.
(CSV)

S2 Table Distances between the sample metatranscriptomes. Spreadsheet containing the Euclidean distances between all 23 samples. Each sample is represented by the normalized log_2_ transcript levels for all 9760 detected genes. The NMDS analysis (Fig 1) was performed on these distances. Treatments in the spreadsheet are ordered by increasing distance to T0 controls, calculated as the median of pairwise distances between treatment and T0 control replicates.
(CSV)

S1 Fig Transcript proportions from major phylogroups. For each major phylogroup and treatment, the mean proportion of transcripts are shown. Transcripts are for all detected genes within the phylogroup and averaged over replicates within the treatment. For each phylogroup the proportions did not vary much across treatments. Consequently, the differential expression we report for a phylogroup mainly reflects changes in how its transcripts were distributed across its gene targets on MicroTOOLs, i.e. up- and down-regulation of its genes. Note that the large proportion of transcripts from *Synechococcus*, which was rare by relative abundance, is because *Synechococcus* has many probes on MicroTOOLs. PS = photosynthetic.
(PDF)

S2 Fig Heat map of differentially expressed genes. A total of 3805 significantly differentially expressed (DE) genes were identified by comparing controls at T0 vs. T24, or treatments with NO3^-^, FDW, or urea at T24 vs. controls at T24. Genes (rows) cluster mainly by phylogroup, evident by the opposite patterns for *Prochlorococcus* and *Synechococcus* genes, mainly for photosynthesis and carbon metabolism. However, also apparent are sub-clusters by Process or Element (e.g., several blocks of N metabolism genes) and by Gene (e.g., *rbcL* across eukaryotic phytoplankton, mostly denoted as “Other”). Sample clusters (columns) were robust (solid discs indicate ≥70% support out of 1000 bootstraps). With the exception of the Fe and NH4^+^ treatments, at least two of the three replicates for each treatment cluster tightly as in Fig 1. White cells indicate that the strain for that gene was not detected in the sample (Materials and methods).
(PDF)

S3 Fig NMDS of CCS and NPSG samples. NMDS of metatranscriptomes from the present CCS study and from an NPSG study [56]. In comparison to the CCS community, the NPSG community shows larger shifts by 24 h in response to added FDW (130 m) that had high concentrations of nitrite. The CCS community appeared to be N-limited (see main text) while the NSPG community was N-starved. Except for unfiltered deep water (UDW), all metatranscriptomes are from surface samples. The *in situ* samples were collected at dawn and dusk from 14–16 September 2011 with an Environmental Sample Processor. In order to compare metatranscriptomes from the CCS and NPSG in a single NMDS analysis, microarray data for samples from both experiments were processed together (including normalization; Materials and methods). The stress was 0.09.
(PDF)

S4 Fig Picocyanobacteria transcript level ratios of *rbcL* to *ntcA*. Ratios of transcript levels for carbon fixation genes (*rbcL*) versus N-stress genes (*ntcA*) are shown for the dominant picocyanobacteria *Prochlorococcus* str. MED4 and *Synechococcus* str. CC9605. For each strain, the *y* axis shows the ratio of the mean transcript levels from detected *rbcL* targets divided by mean transcript levels from detected *ntcA* targets. n = 3 for all ratios for each condition except n = 2 for NH_4_^+^ T24.
(PDF)

S5 Fig Picocyanobacteria responses from Fe stress genes. Conventions are as in Fig 3.
(PDF)

S6 Fig HL *Prochlorococcus* NiSOD. HL *Prochlorococcus* Ni-containing superoxide dismutase (NiSOD) encoding genes. The heat map shows the mean transcript levels (normalized as described in Materials and methods) of all 19 detected NiSOD-encoding genes (“NiSOD targets”, rows) from HL *Prochlorococcus* strains in each treatment. Transcript levels are not centered or scaled. Therefore, deeply red NiSOD targets (e.g. #5590 for MED4) represent sub-populations that were more actively transcribing or were more abundant. For each target gene, transcript levels are ranked across treatments from highest (cells with 1) to lowest (cells with 8). Treatments are sorted by the mean of the ranks in each column: NiSOD targets usually had their highest transcript levels in the T0 controls, second highest levels in the urea treatment, and lowest levels in FDW. Annotation at the left of the heat map indicates for each target gene whether it was DE, the HL strain, and the WGCNA module to which the target was assigned. For targets that were DE in multiple conditions, only one is shown with preference given to treatments over controls. All DE conditions are in S1 Table.
(PDF)

S7 Fig LL *Prochlorococcus* NiSOD. LL *Prochlorococcus* Ni-containing superoxide dismutase genes (*NiSOD*). Heat map conventions are as in S6 Fig.
(PDF)

S8 Fig *Prochlorococcus* MED4 genes *phrB* and *nudix*. Heat map conventions are as in S6 Fig except that gene annotation is included rather than strain because all targets are for MED4.
(PDF)

S9 Fig Picocyanobacteria responses from P stress genes. Conventions are as in Fig 3.
(PDF)

S10 Fig EGSEA results for *Pelagibacter*. Proteorhodopsin genes (*bop*) and iron stress genes (mainly *idiA*) from *Pelagibacter*. These are the main *Pelagibacter* genes represented in MicroTOOLs.
(PDF)

S11 Fig DE genes for *Pelagibacter*. Single-gene DE analysis for *Pelagibacter*. Conventions are as in Fig 3.
(PDF)
