# Supplementary material for: Phytoplankton transcriptomic and physiological responses to fixed nitrogen in the California current system
Source: PLoS One. 2020 Apr 20;15(4):e0231771. doi: 10.1371/journal.pone.0231771 (PMC7170224; doi:10.1371/journal.pone.0231771)
Supplement: S1 File — (DOCX) [file pone.0231771.s001.docx]

**Supporting Information**

**Materials and methods**

**cDNA labeling and hybridization**

cDNA was labeled at the Roy J. Carver Center for Genomics (The University of Iowa, USA) using the Agilent SureTag DNA Labeling Kit (Cat# 5190-3400) and by following a protocol based on Agilent Oligonucleotide Array‐Based CGH for Genomic DNA Analysis: Enzymatic Labeling for Blood, Cells, or Tissues (Version 7.3 March 2014). For each sample, 0.5 µg of cDNA was diluted to 19.2 ng/µl (26.0 µl total), denatured and annealed with 5.0 µl of random primers. The resulting 31.0 µl sample was incubated at 95° C for 5 min, then immediately placed on ice for 5 min, and then centrifuged at 6K × g for 1 min. Nineteen µl of Labeling Master Mix was prepared (10.0 µl of 5X Reaction Buffer, 5.0 µl of 10x dNTP, 3.0 µl cyanine, 1.0 µl Exo-Klenow fragment) and then added into the sample. The sample was briefly centrifuged, then incubated at 37° C for 2 h followed by incubation at 65° C for 10 min and then transferred on ice. Labeled cDNA was then purified using the Agilent SureTag DNA Labeling Kit, which included purification columns. A Nanodrop spectrophotometer was used to check that the yield and specific activity of Cy3 were within expected ranges. Cy3-labeled cDNA was hybridized using a Gene Expression Hybridization Kit (Cat# 5188‐5242) and following the protocol from “One-Color Microarray-Based Gene Expression Analysis: Low Input Quick Amp Labeling (Version 6.7, September 2014).”  For each Cy3-labeled sample, 3.0 µg of DNA was taken, and water added to bring the total volume to 44.00 µl. To each sample, 66.0 µl of hybridization master mix was added (11.0 µl 10x GE blocking agent, 55.0 µl 2x Hi-RPM Hyb Buffer) for a total volume of 110 µl. The sample was mixed, incubated at 95° C for 3 min, placed on ice, and then briefly centrifuged. From each sample, 100 µl was taken and loaded onto one of the 4 arrays on a MicroTOOLs microarray slide. Arrays were hybridized at 65° C for ~18 h. After hybridization, arrays were first washed for 1 min in room temperature Gene Expression Wash Buffer 1 and then washed for 1 min in 37° C Gene Expression Wash Buffer 2 (Gene Expression Wash Buffer Kit, Cat# 5188‐5327).

**Impact of changes in phylogroup transcript relative abundances**

For gene sets that were significantly differentially expressed in the EGSEA analysis, we performed an additional check to see if the differential expression could be attributed solely to a change in the transcript relative abundance of the phylogroup in the two samples being compared. For example, we checked whether increases in HL *Prochlorococcus* photosynthesis transcripts in NH_4_^+^ vs. controls at T24 reflected an overall increase in transcripts from HL *Prochlorococcus*, perhaps due to an overall decrease in transcripts from all other phylogroups (S1 Fig). Each phylogroup’s total transcript relative abundance varied little across the samples (S1 Fig) and had fold changes that were always less than the transcript level fold changes for gene sets reported as differentially expressed, with the exception of eukaryote *rbcL* in FDW. However, our check is more useful for phylogroups with a broad set of genes on MicroTOOLs to anchor the total transcripts for the phylogroup, whereas eukaryotes are almost exclusively represented by *rbcL*.

**Results and discussion**

**Metatranscriptome interpretation based on NMDS clusters**

NMDS clusters were strongly influenced by changes in transcript levels from HL *Prochlorococcus*, *Synechococcus*, and photosynthetic eukaryotes (PE; Fig 1) because these phylogroups are highly represented in the MicroTOOLs microarray design [1]. Across T24 samples, the total proportion of transcripts attributed to each phylogroup was stable (max s.d. 0.05, for HL *Prochlorococcus*; S1 Fig), as were relative abundances [2]. Thus, the metatranscriptome positions in Fig 1 reflect changes in the distribution of transcripts within each of these phylogroups. Note that heterotrophic bacteria were also abundant, and those with larger genomes (HNA) underwent large cell density increases in all treatments [2]. However, heterotrophs have fewer MicroTOOLs targets and thus had limited influence in the NMDS analysis. In contrast, although PE are represented mainly by *rbcL* in MicroTOOLs, there are many *rbcL* targets.

Although transcript level changes from N stress genes (described in the main text) indicated that the CCS surface phytoplankton community was N-limited, in comparison to the NSPG, the CCS community as detected with the microarray either was not N-starved or was co-limited by N and another nutrient. This is supported by the significantly tighter clustering of the CCS versus the NPSG metatranscriptomes (p~0 in a PERMDISP2 [3] analysis using the vegan R package function betadisper on Euclidean distances between samples followed by ANOVA) apparent in S3 Fig and also by the single-gene and EGSEA analyses (main text). An alternative explanation for the clustering differences is that higher diversity in the CCS might have caused transcripts to be distributed over more MicroTOOLs targets, and thus limited the influence of transcriptional changes from any single phylogroup in the CCS metatranscriptomes. In particular, across CCS samples, *Prochlorococcus* versus *Synechococcus* transcripts were ~1:1 (the ratio of the medians of each phylogroup proportion of transcripts) but ~2:1 across NPSG samples in the study by Robidart et al. [4]. Most likely, nutrient statuses and diversity both influenced the clustering differences between CCS and NPSG metatranscriptomes.

The high diversity of the microbial community at the CCS station also may have contributed to the higher variation among replicate metatranscriptomes, such as the outlier N+Fe sample (Fig 1). This sample had the smallest proportion of transcripts from viruses (6.7% vs. 11.6±1.5% for all samples) and, perhaps consequently, the largest proportion of transcripts from HL *Prochlorococcus* (42.2% vs. 31.4±5.1% for all samples). It is possible that other replicates that clustered poorly (e.g. the outlier Fe sample) differed with respect to viruses or grazers not represented on MicroTOOLs. High variation among replicate metatranscriptomes from coastal communities in comparison to oligotrophic communities have been observed in other metatranscriptomic studies [5,6].

**Changes in ratios of *rbcL* to *ntcA* transcript levels in *Prochlorococcus* and *Synechococcus***

As a simple proxy for cellular needs of carbon (C) versus N, we examined relative transcript levels for *rbcL* versus *ntcA* for the dominant HL strain MED4. We hypothesized that a high C:N ratio, indicating N limitation, would lead to increased transcription of *ntcA* to acquire N rather than to increased transcription of *rbcL* to fix CO_2_. The *rbcL* to *ntcA* ratio in *Prochlorococcus* increased in all N treatments, and the highest increases were in the urea, NO_3_^-^, and NH_4_^+^ treatments in comparison to controls and all other treatments at T24 (not significant [p>0.01] in Mann-Whitney U test, with just 2-3 replicate ratios in each comparison; S4 Fig). The similar results in urea and NH_4_^+^ suggested that both substrates provided enough N to shift the internal C-N balance in *Prochlorococcus* MED4 within 24 hours.

The most abundant *Synechococcus* strain CC9605 had small changes in the ratio of *rbcL* to *ntcA* in controls and all treatments at T24 (1.1±0.35) in comparison to *Prochlorococcus* (4.6±3.8; S4 Fig). For both genera, the most elevated ratios were observed in the treatments with NH_4_^+^, NO_3_^-^, and urea. In these treatments both *Prochlorococcus* and *Synechococcus* cell densities increased significantly but modestly by T48 in comparison to controls, except for NH_4_^+^ where *Synechococcus* cell abundances decreased (Fig 2B). This suggests that *Synechococcus* was at a disadvantage or used a different metabolic strategy compared to *Prochlorococcus* in utilizing added NH_4_^+^.

**Transcriptionally active *Prochlorococcus* strains and oligotypes detected by 16S-rRNA gene analysis**

The subpopulations of *Prochlorococcus* MIT9515 and MIT9301 that had different responses to NO_3_^-^ might correspond to oligotypes defined by Shilova et al., [2] based on 16S rRNA gene sequence analysis [2,7]. In T0 samples, two main oligotypes were identified for MIT9515 (10.72% and 1.42% of total *Prochlorococcus*) and also for MIT9301 (relative abundances of both ≤ 0.29% of total *Prochlorococcus*). However, we have no genomic sequence data to link oligotypes to the PS genes, and it is possible that the detected PS transcripts from these natural populations were similar to MIT9515 and MIT9301 only with respect to these genes.

***Prochlorococcus* responses from other stress genes**

Several *Prochlorococcus* genes associated with reactive oxygen species and light stress responded to the treatments. In the NO_3_^-^ and FDW treatments, transcript levels from genes that encode NiSOD, a putative nickel-containing superoxide dismutase precursor, decreased for HL strains (DE for MED4 and MIT9515; S6 Fig) but increased for LL strains (DE for CCMP1375 and MIT9313; S7 Fig). EGSEA corroborated the NiSOD gene increases from LL strains in response to FDW. EGSEA also indicated that HL *Prochlorococcus* genes associated with light stress (UV damage or subsequent DNA repair) decreased in all treatments, except they increased in N+Fe. These genes included *phrB* which encodes a DNA photolyase, *nudix* which encodes nudix hydroxylase, and *pmm1359* whose function is not known (but highly light-responsive, [8]. S8 Fig illustrates for *phrB* and *nudix* targets for MED4 the EGSEA result, with most targets having lower transcript levels relative to controls at T24 for all treatments except N+Fe.

***Synechococcus* responses from genes associated with P, Fe, and other stresses**

For some *Synechococcus*, increases in available Fe may have resulted in transcription increases for genes associated with P stress. The EGSEA analysis showed overall transcript level increases for P stress genes in response to the addition of Fe, N+Fe, and FDW (with an unknown concentration of Fe) but not in response to treatments that added only N (Fig 2B). However, the single-gene DE analysis revealed strain- and gene-specific responses (S9 Fig). For example, in the NO_3_^-^ treatment, *psiP* (P-stress inducible protein) transcripts increased for strain WH8102 (clade III), but phosphate transporters (*pstS*) decreased for the dominant strain CC9902 and for RS9916. In the FDW treatment as well, WH8102 and RS9916 had opposite responses for, respectively, *psiP* (increased) and *pstS* (decreased).

*Synechococcus* genes that are usually elevated during Fe limitation increased in response to FDW and N+Fe (Fig 2B), but once again, responses differed among strains and genes (S5 Fig). In response to FDW, strain-specific EGSEA analyses found that transcript levels from Fe stress genes increased >1.2× on average for strains CC9311 (*dpsA* was DE), RCC307, WH7803, and CC9902. However, for CC9902 one *fur* target decreased DE in response to FDW (S5 Fig). This same target also decreased DE in response to NO_3_^-^, along with a third *fur* target. Finally, EGSEA showed transcript level increases from RCC307 iron stress genes in response to treatments with Fe (alone or N+Fe).

As with HL *Prochlorococcus*, the addition of N+Fe led to overall transcript level increases for *Synechococcus* genes associated with light stress (or associated DNA repair genes; *phrB*, *nudix*, and *pmm1359* in the EGSEA “light stress” gene set).

**Correlated transcription of picocyanobacteria urea uptake genes**

The weighted correlation network analysis (WGCNA) assigned most (75%) picocyanobacteria *urtA* genes to the same module indicating significantly correlated transcript levels across the samples. WGCNA module M10 was comprised almost exclusively of picocyanobacteria *urtA* genes (118 of 140 genes in M10; S1 Table). These included 109 of 133 detected *Prochlorococcus* *urtA* genes for multiple strains from HL and LL ecotypes. Most other *Prochlorococcus* genes associated with N stress or metabolism were assigned to M1 (295 of 367 detected). Similarly, M10 had the most *Synechococcus urtA* genes (9 of 25 detected) while most of the other 462 detected *Synechococcus* N genes were assigned to modules M1 and M3 (92 and 93 targets, respectively).

**Transcription changes among *Pelagibacter***

Transcripts from *Pelagibacter ubique* spp. HTCC7211, HTCC1062 and HTCC1002 were detected in every sample (from 41%, 13%, and 16% of their respective MicroTOOLs targets; S1 Table), consistent with the stable relative abundances of *Pelagibacter* from T0 to T24 in controls and treatments [2]. Transcript level changes for *Pelagibacter* proteorhodopsin genes (*bop*) differed across treatments. For example, the EGSEA analysis found that *bop* transcript levels increased in response to NH_4_^+^ but decreased in response to Fe, N+Fe, or FDW (S10 Fig). We interpret the *bop* increases in the NH_4_^+^ treatment (mainly from strain HTCC7211) as a response to carbon (C) starvation [9] because at T48 Shilova et al. [2] observed the second highest primary productivity rates and Chl *a* concentration, as well as highest cell abundances for HNA cells and *Prochlorococcus* and the lowest cell abundances for LNA cells (which include *Pelagibacter ubique*). The *bop* decreases in response to Fe-containing treatments have, to our knowledge, not been reported for *Pelagibacter ubique*, however, proteorhodopsin transcription increases under Fe limitation have been observed for some diatoms [10] and suspected for pelagophytes and dinoflagellates [11]. We observed strain-specific responses in other treatments as well. For example, in response to FDW, large (>1.2×) *bop* transcript level decreases occurred for strains HTCC7211 and HTCC1002, and also for alphaproteobacterium HIMB5, but HTCC1062 did not respond to FDW (S10 Fig, S11 Fig). Single-gene analysis showed that in response to NO_3_^-^, *bop* transcript levels increased DE for HTCC1062 but decreased DE for HIMB5 and HTCC7211 (with one exception for HTCC7211 that increased).

In the EGSEA analysis, transcript levels from *Pelagibacter* Fe stress genes had large decreases in response to FDW or NO_3_^-^, and weak (<1.2×) decreases in response to urea or Fe (S10 Fig). Strain HTCC7211 likely accounted for the decreases because it has the most MicroTOOLs Fe gene targets within *Pelagibacter*, including 171 targets for iron transporter *idiA*. The results were similar from the single-gene DE analysis with decreased *idiA* transcript levels in FDW and NO_3_^-^ (S11 Fig).

Treatments may have affected *Pelagibacter* metabolism of dimethyl sulfoniopropionate (DMSP) based on transcript level changes for *dmdA* which encodes a demethylase for DMSP. Treatments with NO_3_^-^ or with FDW, which had the same concentration of NO_3_^-^, resulted in transcript level decreases for some *Pelagibacter* targets for *dmdA*. Large (>1.2×) decreases occurred for *Pelagibacter* spp. HTCC7211 in response to NO_3_^-^ (strain-specific EGSEA analysis), and DE decreases were observed for targets from HTCC7211 and HTCC1002 in response to FDW (S11 Fig, S1 Table). We note that EGSEA did not indicate increases (or changes) for *dmdA* in the NH_4_^+^ treatment, neither overall for *Pelagibacter* nor individually for strains HTCC7211, HTCC1002, or HTCC1062. Thus, although DMSP is known to be a C source for marine bacteria [12,13], DMSP was not likely utilized to address the C starvation hypothesized in the NH_4_^+^ treatment.

**References**

1. Shilova IN, Robidart JC, James Tripp H, Turk-Kubo K, Wawrik B, Post AF, et al. A microarray for assessing transcription from pelagic marine microbial taxa. ISME J. 2014 Jul;8(7):1476–91.

2. Shilova IN, Mills MM, Robidart JC, Turk-Kubo KA, Björkman KM, Kolber Z, et al. Differential effects of nitrate, ammonium, and urea as N sources for microbial communities in the North Pacific Ocean. Limnol Oceanogr. 2017 Nov;62(6):2550–74.

3. Anderson MJ. Distance-based tests for homogeneity of multivariate dispersions. Biometrics. 2006 Mar;62(1):245–53.

4. Robidart JC, Magasin JD, Shilova IN, Turk-Kubo KA, Wilson ST, Karl DM, et al. Effects of nutrient enrichment on surface microbial community gene expression in the oligotrophic North Pacific Subtropical Gyre. ISME J. 2018 Sep 1;13(2):374–87.

5. Poretsky RS, Hewson I, Sun S, Allen AE, Zehr JP, Moran MA. Comparative day/night metatranscriptomic analysis of microbial communities in the North Pacific Subtropical Gyre. Environ Microbiol. 2009 Jun;11(6):1358–75.

6. Shi Y, McCarren J, DeLong EF. Transcriptional responses of surface water marine microbial assemblages to deep-sea water amendment: microbial transcriptome responses to deep-water addition. Environ Microbiol. 2012 Jan;14(1):191–206.

7. Eren AM, Maignien L, Sul WJ, Murphy LG, Grim SL, Morrison HG, et al. Oligotyping: differentiating between closely related microbial taxa using 16S rRNA gene data. Methods Ecol Evol. 2013;4(12):1111–9.

8. Thompson AW, Huang K, Saito MA, Chisholm SW. Transcriptome response of high- and low-light-adapted *Prochlorococcus* strains to changing iron availability. ISME J. 2011 Oct;5(10):1580–94.

9. Steindler L, Schwalbach MS, Smith DP, Chan F, Giovannoni SJ. Energy starved *Candidatus* Pelagibacter ubique substitutes light-mediated ATP production for endogenous carbon respiration. PLOS ONE. 2011 May 9;6(5):e19725.

10. Marchetti A, Catlett D, Hopkinson BM, Ellis K, Cassar N. Marine diatom proteorhodopsins and their potential role in coping with low iron availability. ISME J. 2015 Dec;9(12):2745–8.

11. Caputi L, Carradec Q, Eveillard D, Kirilovsky A, Pelletier E, Karlusich JJP, et al. Community-level responses to iron availability in open ocean plankton ecosystems. Glob Biogeochem Cycles. 2019;33(3):391–419.

12. Kiene RP, Linn LJ. Distribution and turnover of dissolved DMSP and its relationship with bacterial production and dimethylsulfide in the Gulf of Mexico. Limnol Oceanogr. 2000;45(4):849–61.

13. Simó R, Archer SD, Pedrós-Alió C, Gilpin L, Stelfox-Widdicombe CE. Coupled dynamics of dimethylsulfoniopropionate and dimethylsulfide cycling and the microbial food web in surface waters of the North Atlantic. Limnol Oceanogr. 2002;47(1):53–61.
