# Supplementary figures and images for: Phytoplankton transcriptomic and physiological responses to fixed nitrogen in the California current system
Source: PLoS One. 2020 Apr 20;15(4):e0231771. doi: 10.1371/journal.pone.0231771 (PMC7170224; doi:10.1371/journal.pone.0231771)

Proportion of transcripts

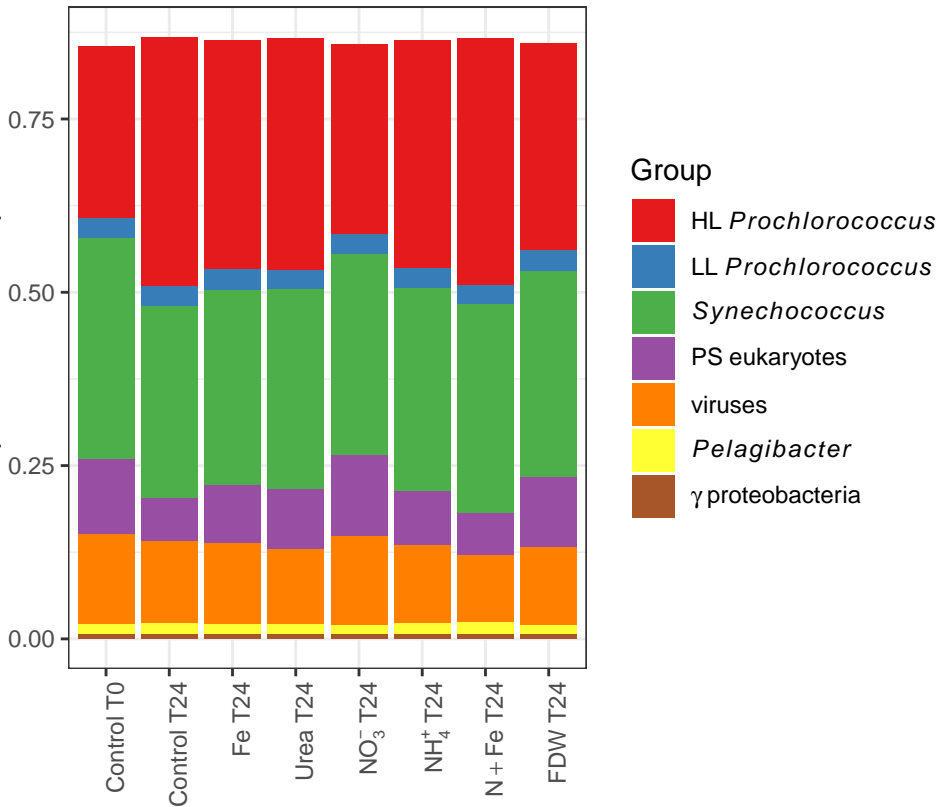

Supplement: S1 Fig — For each major phylogroup and treatment, the mean proportion of transcripts are shown. Transcripts are for all detected genes within the phylogroup and averaged over replicates within the treatment. For each phylogroup the proportions did not vary much across treatments. Consequently, the differential expression we report for a phylogroup mainly reflects changes in how its transcripts were distributed across its gene targets on MicroTOOLs, i.e. up- and down-regulation of its genes. Note that the large proportion of transcripts from Synechococcus, which was rare by relative abundance, is because Synechococcus has many probes on MicroTOOLs. PS = photosynthetic. (PDF) [file pone.0231771.s004.pdf]

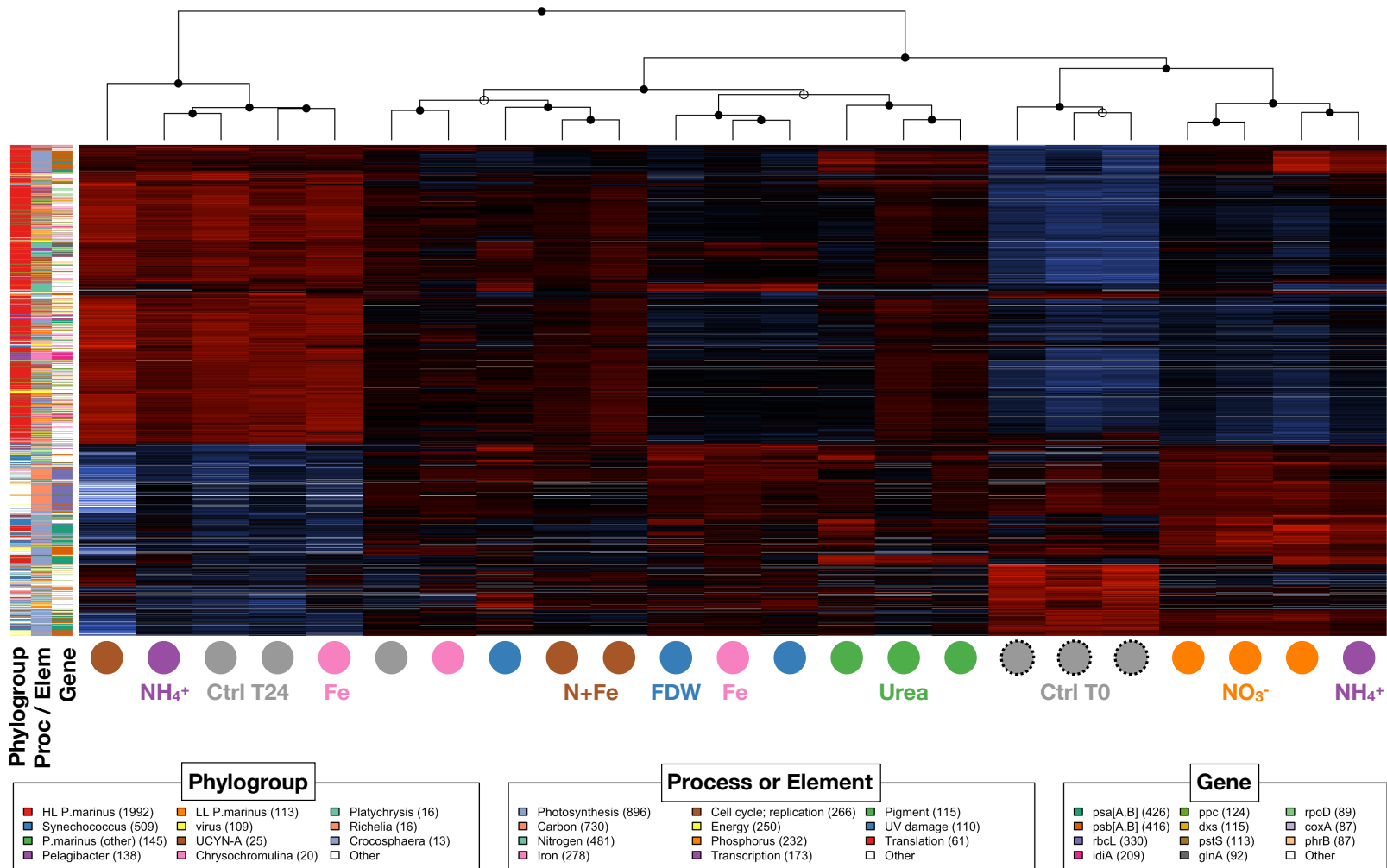

Supplement: S2 Fig — A total of 3805 significantly differentially expressed (DE) genes were identified by comparing controls at T0 vs. T24, or treatments with NO3-, FDW, or urea at T24 vs. controls at T24. Genes (rows) cluster mainly by phylogroup, evident by the opposite patterns for Prochlorococcus and Synechococcus genes, mainly for photosynthesis and carbon metabolism. However, also apparent are sub-clusters by Process or Element (e.g., several blocks of N metabolism genes) and by Gene (e.g., rbcL across eukaryotic phytoplankton, mostly denoted as “Other”). Sample clusters (columns) were robust (solid discs indicate ≥70% support out of 1000 bootstraps). With the exception of the Fe and NH4+ treatments, at least two of the three replicates for each treatment cluster tightly as in Fig 1. White cells indicate that the strain for that gene was not detected in the sample (Materials and methods). (PDF) [file pone.0231771.s005.pdf]

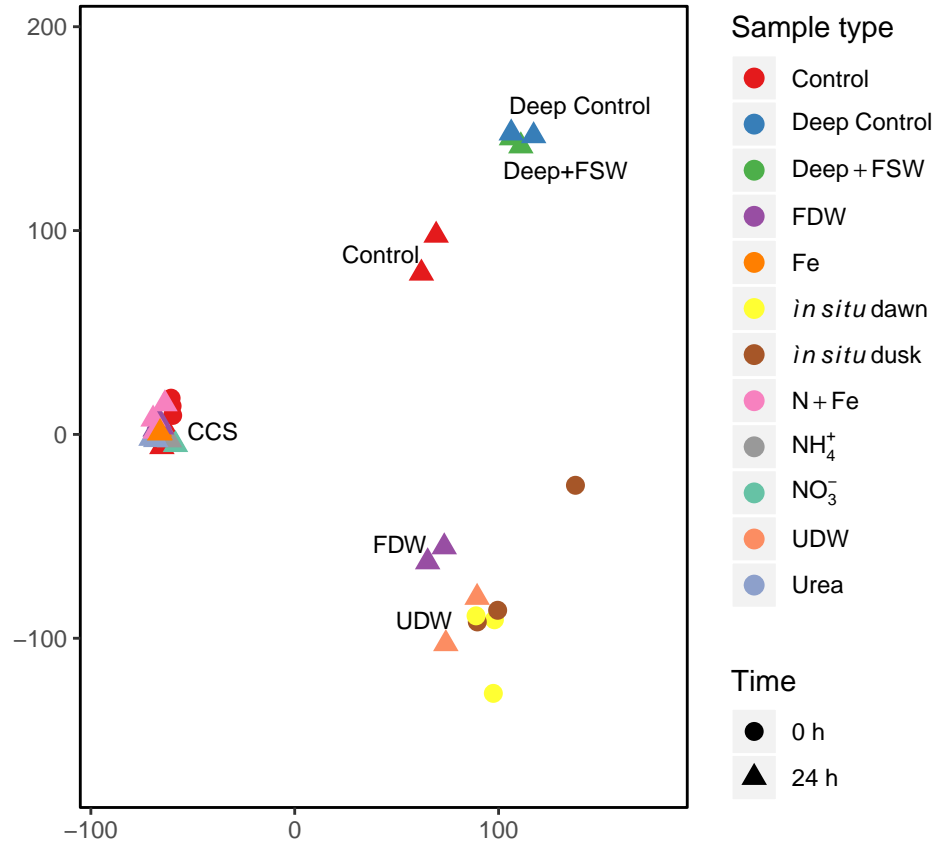

Supplement: S3 Fig — NMDS of metatranscriptomes from the present CCS study and from an NPSG study [56]. In comparison to the CCS community, the NPSG community shows larger shifts by 24 h in response to added FDW (130 m) that had high concentrations of nitrite. The CCS community appeared to be N-limited (see main text) while the NSPG community was N-starved. Except for unfiltered deep water (UDW), all metatranscriptomes are from surface samples. The in situ samples were collected at dawn and dusk from 14–16 September 2011 with an Environmental Sample Processor. In order to compare metatranscriptomes from the CCS and NPSG in a single NMDS analysis, microarray data for samples from both experiments were processed together (including normalization; Materials and methods). The stress was 0.09. (PDF) [file pone.0231771.s006.pdf]

Ratio of *rbcL* to *ntcA* transcript levels

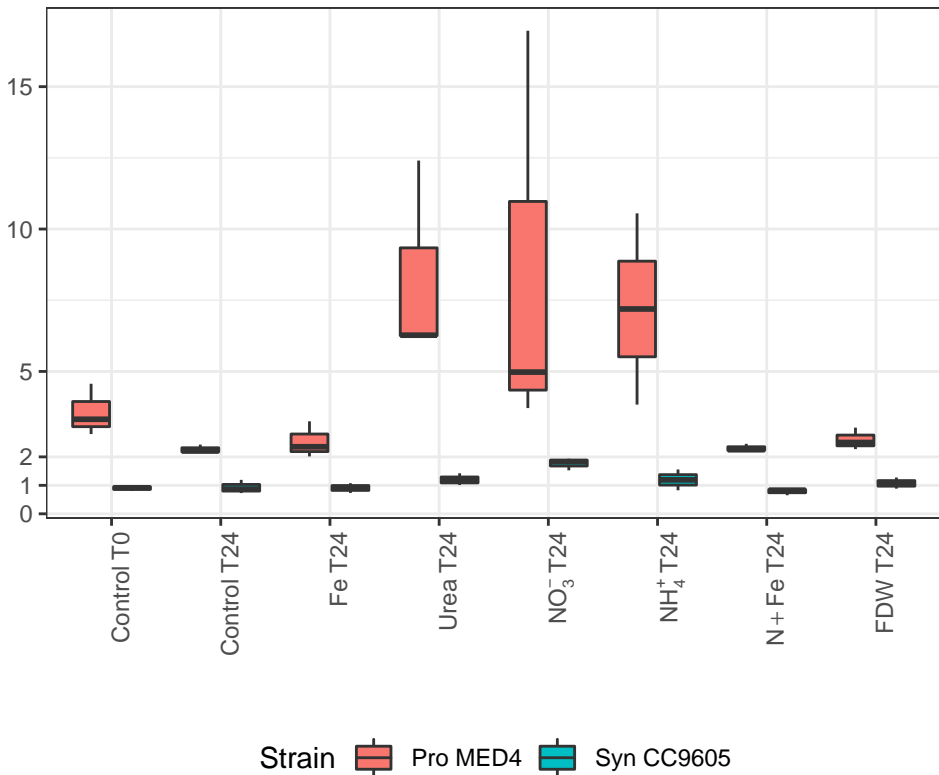

Supplement: S4 Fig — Ratios of transcript levels for carbon fixation genes (rbcL) versus N-stress genes (ntcA) are shown for the dominant picocyanobacteria Prochlorococcus str. MED4 and Synechococcus str. CC9605. For each strain, the y axis shows the ratio of the mean transcript levels from detected rbcL targets divided by mean transcript levels from detected ntcA targets. n = 3 for all ratios for each condition except n = 2 for NH4+ T24. (PDF) [file pone.0231771.s007.pdf]

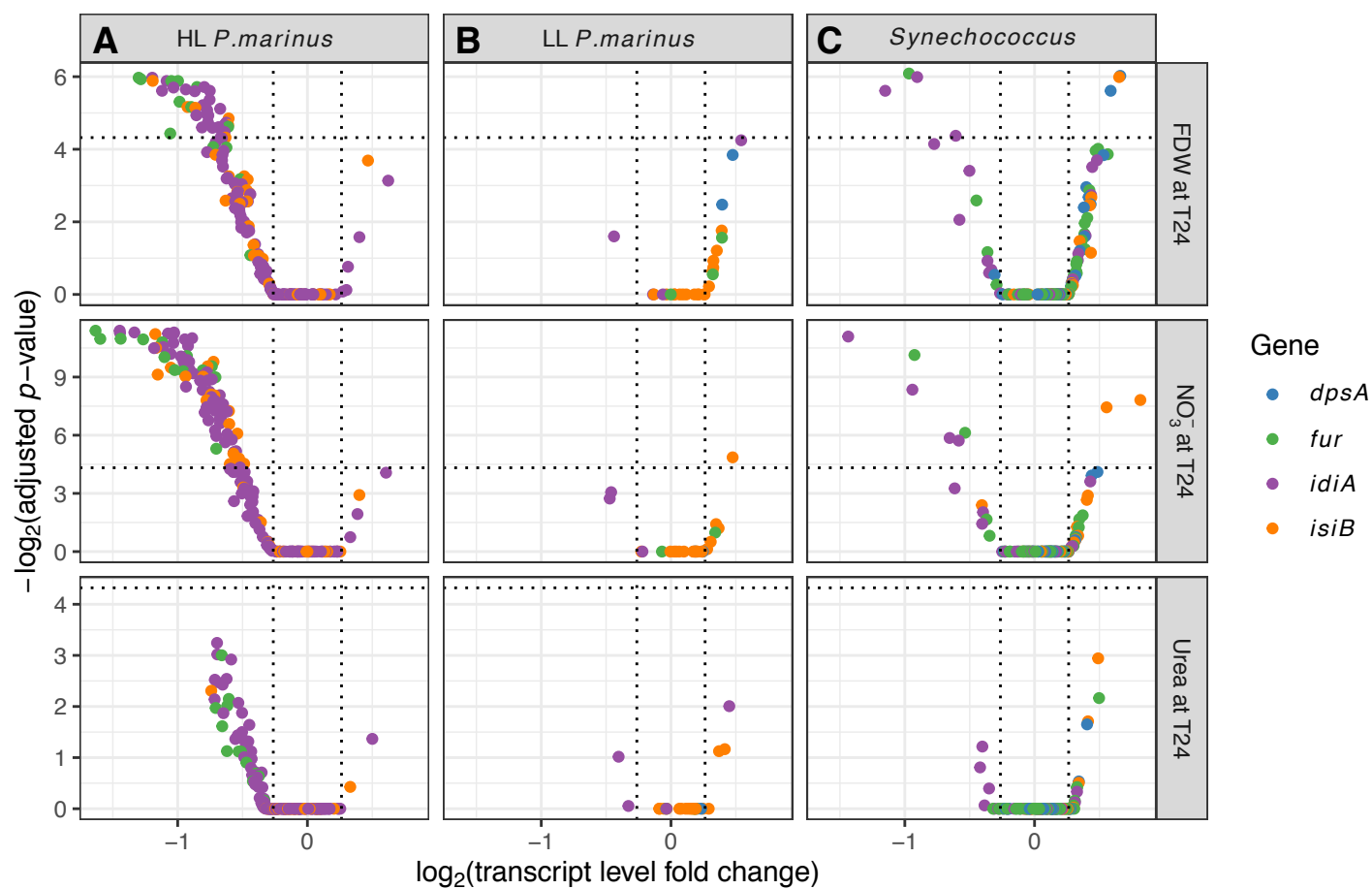

Supplement: S5 Fig — Conventions are as in Fig 3. (PDF) [file pone.0231771.s008.pdf]

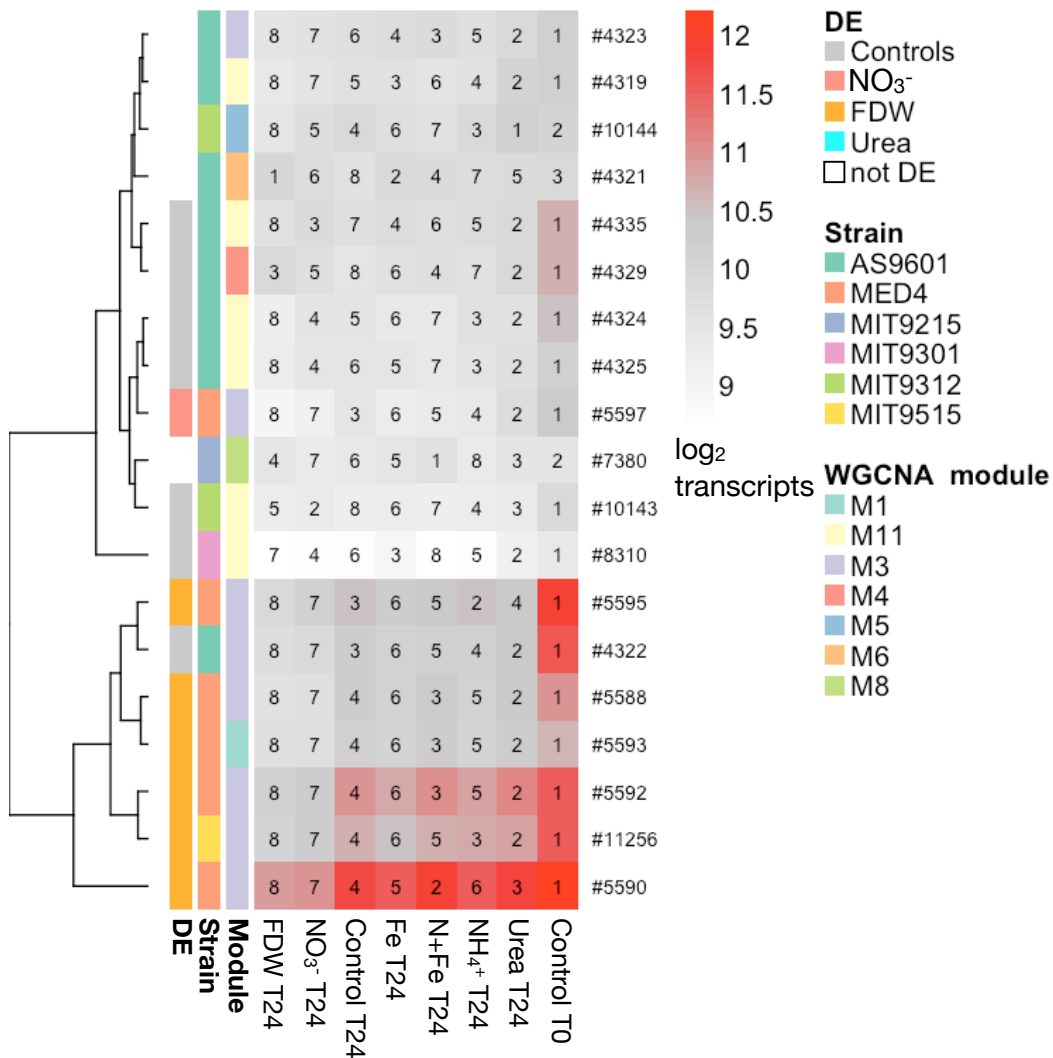

Supplement: S6 Fig — HL Prochlorococcus Ni-containing superoxide dismutase (NiSOD) encoding genes. The heat map shows the mean transcript levels (normalized as described in Materials and methods) of all 19 detected NiSOD-encoding genes (“NiSOD targets”, rows) from HL Prochlorococcus strains in each treatment. Transcript levels are not centered or scaled. Therefore, deeply red NiSOD targets (e.g. #5590 for MED4) represent sub-populations that were more actively transcribing or were more abundant. For each target gene, transcript levels are ranked across treatments from highest (cells with 1) to lowest (cells with 8). Treatments are sorted by the mean of the ranks in each column: NiSOD targets usually had their highest transcript levels in the T0 controls, second highest levels in the urea treatment, and lowest levels in FDW. Annotation at the left of the heat map indicates for each target gene whether it was DE, the HL strain, and the WGCNA module to which the target was assigned. For targets that were DE in multiple conditions, only one is shown with preference given to treatments over controls. All DE conditions are in S1 Table. (PDF) [file pone.0231771.s009.pdf]

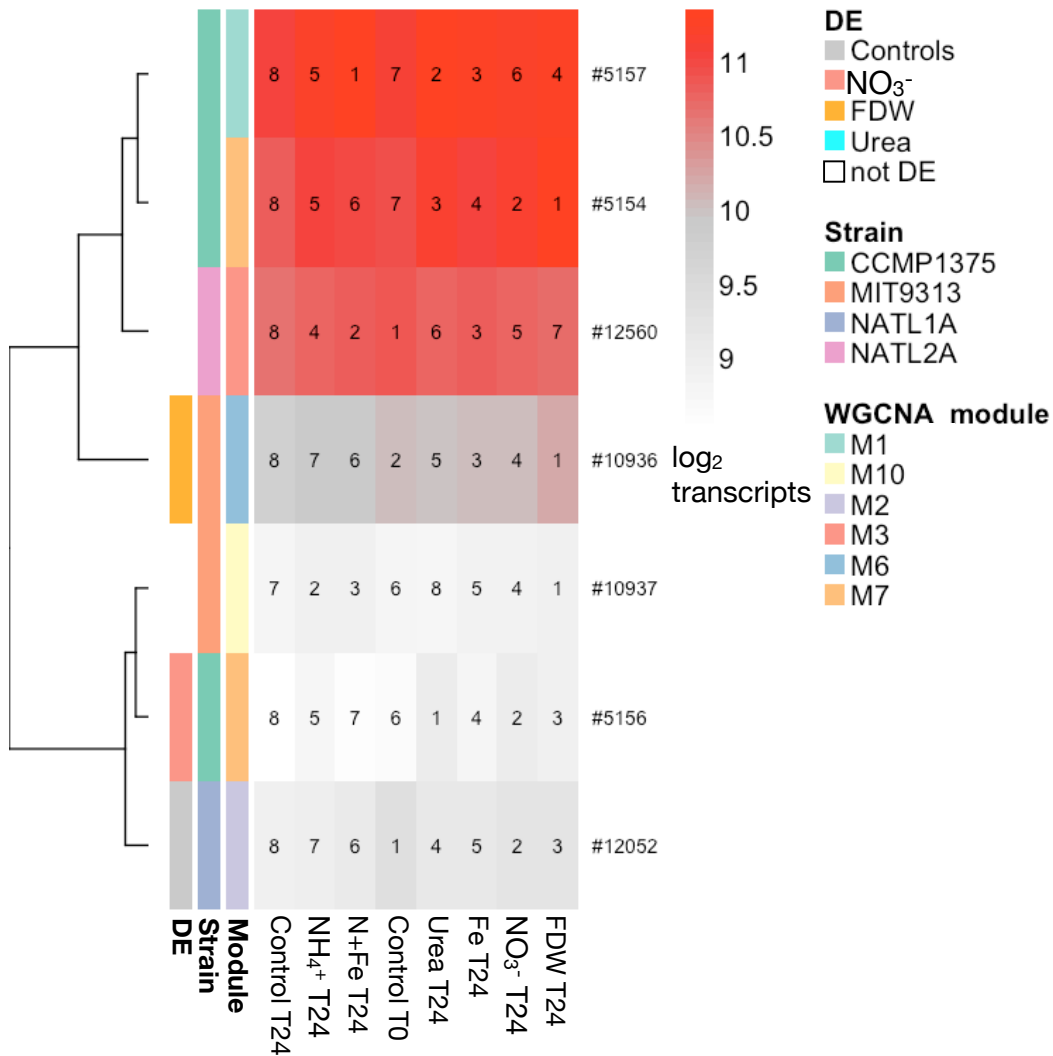

Supplement: S7 Fig — LL Prochlorococcus Ni-containing superoxide dismutase genes (NiSOD). Heat map conventions are as in S6 Fig. (PDF) [file pone.0231771.s010.pdf]

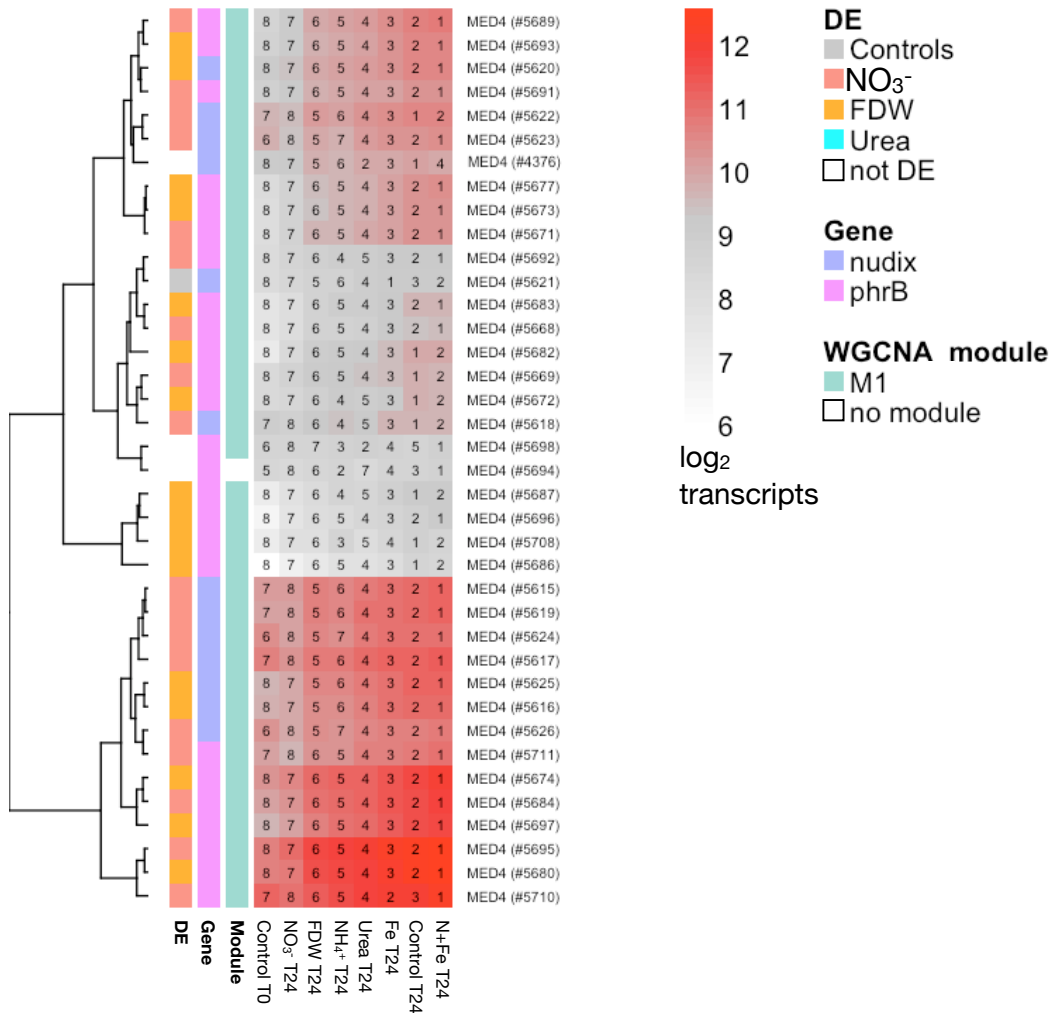

Supplement: S8 Fig — Heat map conventions are as in S6 Fig except that gene annotation is included rather than strain because all targets are for MED4. (PDF) [file pone.0231771.s011.pdf]

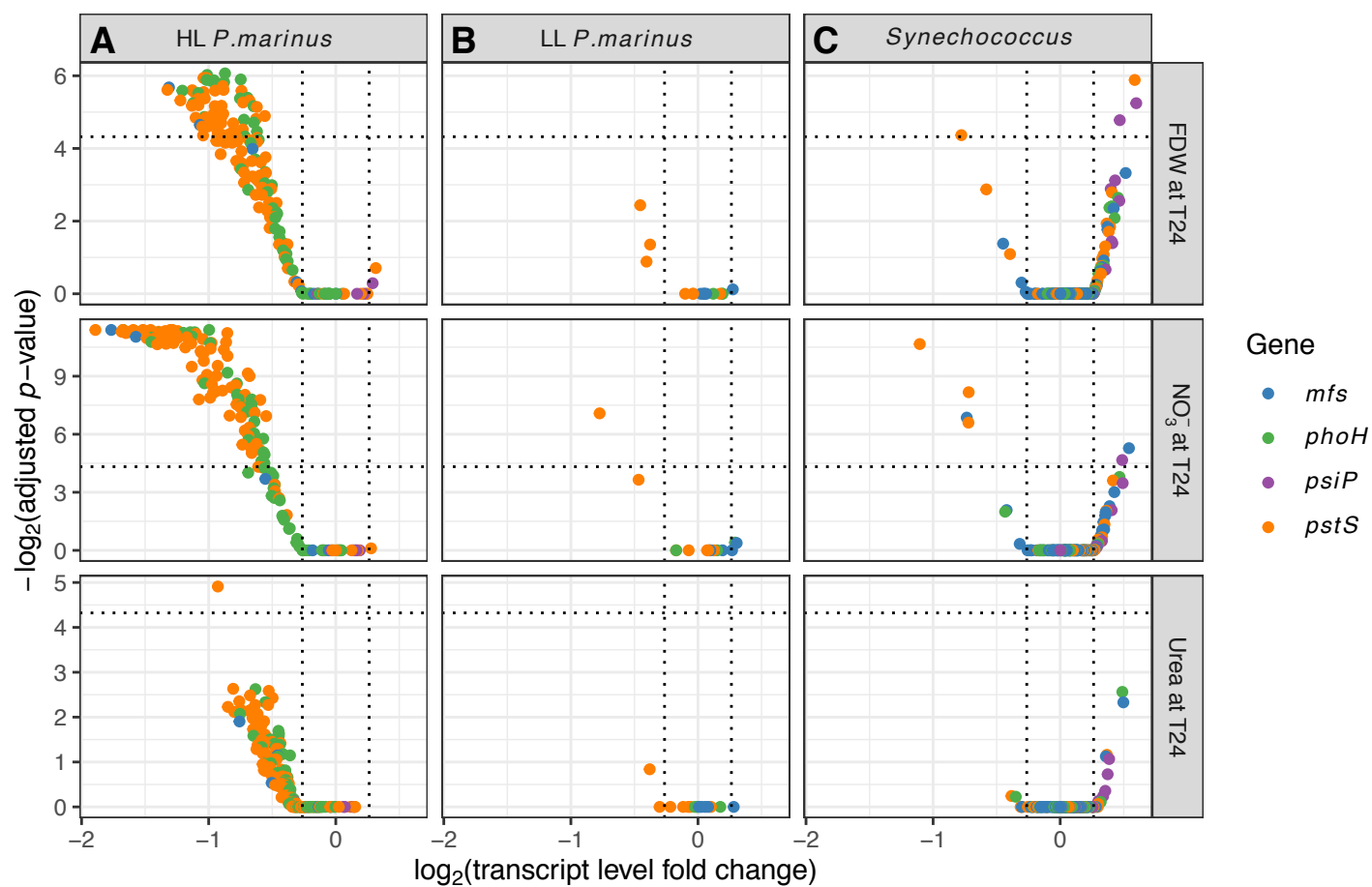

Supplement: S9 Fig — Conventions are as in Fig 3. (PDF) [file pone.0231771.s012.pdf]

# *Pelagibacter*

| Gene set   | Ctrl | Fe | Urea | NO <sub>3</sub> <sup>-</sup> | NH <sub>4</sub> <sup>+</sup> | N+Fe | FDW |
|------------|------|----|------|------------------------------|------------------------------|------|-----|
| <i>bop</i> | ○    | ↓  | ○    | ○                            | ↑                            | ↓    | ↓   |
| Fe stress  | ↑    | ↓  | ↓    | ↓                            | ○                            | ○    | ↓   |

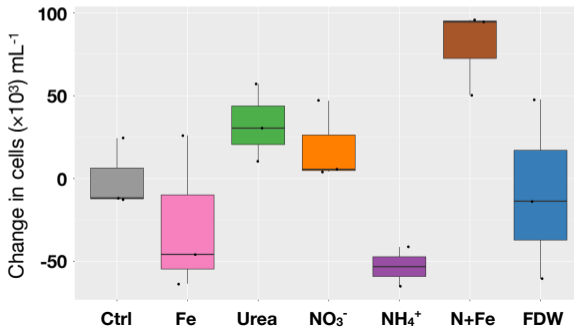

Supplement: S10 Fig — Proteorhodopsin genes (bop) and iron stress genes (mainly idiA) from Pelagibacter. These are the main Pelagibacter genes represented in MicroTOOLs. (PDF) [file pone.0231771.s013.pdf]

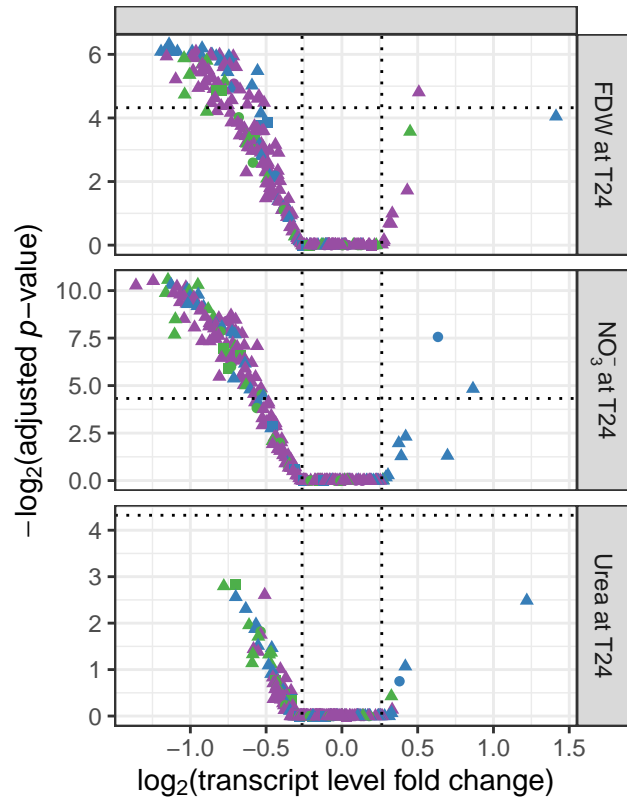

Supplement: S11 Fig — Single-gene DE analysis for Pelagibacter. Conventions are as in Fig 3. (PDF) [file pone.0231771.s014.pdf]
